# Information and communications technology use to prevent and respond to sexual and gender‐based violence in low‐ and middle‐income countries: An evidence and gap map

**DOI:** 10.1002/cl2.1277

**Published:** 2022-10-25

**Authors:** William Philbrick, Jacob Milnor, Madhu Deshmukh, Patricia Mechael

**Affiliations:** ^1^ Sitara International Atlanta Georgia USA; ^2^ Health, Equity and Rights, CARE Atlanta Georgia USA; ^3^ Oswaldo Cruz Foundation Oswaldo Cruz Institute Rio de Janeiro Brazil; ^4^ CARE Atlanta Georgia USA; ^5^ HealthEnabled Washington District of Columbia USA

## Abstract

**Background:**

The use of information and communications technologies (ICT) in low‐ and middle‐income countries (LMIC) has increased significantly in the last several years, particularly in health, including related areas such as preventing and responding to sexual and gender‐based violence (SGBV) against women and children. While the evidence for ICT effectiveness has grown significantly in the past 5 years in other aspects of health, it has not for effectiveness of using ICT for the prevention and response to SGBV against women and children in LMIC.

**Objectives:**

The primary goal of this evidence and gap map (EGM) is to establish a baseline for the state of the evidence connected with the use of ICT for preventing and responding to SGBV against women and children in LMIC. Objectives that contribute to the achievement of this goal are:

(1) identifying evidence of effectiveness for the use of ICT targeting the prevention of, and response to, SGBV against women and children in LMIC;
(2) identifying key gaps in the available ICT for SGBV prevention and/or response evidence;
(3) identifying research methodology issues reflected in the current evidence;
(4) identifying any clusters of evidence in one or more ICT interventions suitable for systematic review;
(5) identifying enabling factors associated with effective interventions using ICT for the prevention of, and response to, SGBV against women and children in LMIC; and
(6) providing a structured and accessible guide to stakeholders for future investment into interventions and research using ICT for SGBV prevention and response in LMIC.

**Search Methods:**

The date of the last search from which records were evaluated, and any studies identified were incorporated into the EGM was July 11, 2021. Twenty (20) databases were searched, and identified under “Methods.”

**Selection Criteria:**

We conducted systematic searches of multiple academic databases using search terms and criteria related to the use of ICT for prevention and/or response to SGBV against women and children. Although excluded, we did consider studies conducted in higher‐income countries (HIC) only to provide context and contrast for the EGM discussion of the eligible studies from LMIC.

**Data Collection and Analysis:**

The EGM search process included five phases: (1) initial search of academic databases conducted by two researchers simultaneously; (2) comparison of search results, and abstract screening by two researchers collaboratively; (3) second screening by reviewing full articles of the studies identified in the first screening by two reviewers independently; (4) comparison of results of second screening; resolution of discrepancies of screening results; and (5) data extraction and analysis.

**Main Results:**

The EGM includes 10 studies published in English of which 4 were systematic, literature or scoping reviews directly addressing some aspect of the use of ICT for SGBV prevention and/or response in women and girls. The six individual studies were, or are being, conducted in LMIC (a condition for eligibility). No eligible studies addressed children as a target group, although a number of the ineligible studies reported on the use of ICT for intermediate outcomes connected with violence against children (e.g., digital parenting). Yet, such studies did not explicitly attach those intermediate outcomes to SGBV prevention or response outcomes. Countries represented among the eligible individual studies include Cambodia, Kenya, Nepal Democratic Republic of Congo (DRC), and Lebanon. Of the 10 eligible studies (individual and reviews), most focused on intimate partner violence against women (IPV). Intervention areas among the eligible studies include safety planning using decision algorithms, educational and empowerment messaging regarding norms and attitudes towards gender‐based violence (GBV), multi‐media radio drama for social behavior change, the collection of survivor experience to inform SGBV/GBV services, and the collection of forensic evidence connected to the perpetration of SGBV. Thirty‐one studies which otherwise would have been eligible for the evidence and gap map (EGM) were conducted in HIC (identified under “Excluded Reviews”). None of the eligible studies reported results related to effectiveness of using ICT in a control setting, for the primary prevention of SGBV as an outcome, but rather reported on outcomes such as usability, secondary and tertiary prevention, feasibility, access to services and other outcomes primarily relating to the development of the interventions. Two studies identified IPV prevention as a measurable outcome within their protocols, but one of these had not yet formally published results regarding primary prevention as an outcome. The other study, while reporting on the protocol (and steps to adapt the ICT application, previously reported as effective in HIC contexts to a specific LMIC context), has not yet as of the date of writing this EGM, published outcome results related to the reduction of IPV. Of the four reviews identified as eligible, two are better characterized as either a literature review or case study rather than as traditional systematic reviews reporting on impact outcomes with methodologically rigorous protocols.

**Authors' Conclusions:**

The evidence baseline for using ICT to prevent and/or respond to SGBV against women and children in LMIC is nascent. Promising areas for future study include: (1) how ICT can contribute changing gender and social norms related to SGBV and primary prevention; (2) mobile phone applications that promote safety and security; (3) mobile technology for the collection and analysis of survivors' experience with SGBV response services; and (4) digital tools that support the collection of forensic evidence for SGBV response and secondary prevention. Most striking is the paucity of eligible studies examining the use of ICT in connection with preventing or responding to SGBV against children. In light of the exponential increase in the use of ICT by children and adolescents, even in LMIC, greater attention should be given to examining how ICT can be used during adolescence to address gender norms that lead to SGBV. While there appears to be interest in using ICT for SGBV prevention and/or response in LMIC, other than several ad hoc studies, there is little evidence of if, and *how effective* these interventions are. Further inquiry should be made regarding if and how interventions proven effective in HIC can be adapted to LMIC contexts.

## 1 PLAIN LANGUAGE SUMMARY

### 1.1 There is a small but growing evidence base underlying ICT interventions for prevention and response to sexual and gender‐based violence in low‐ and middle‐income countries

Methodologically rigorous evidence examining the effectiveness of information and communication technology (ICT) in preventing and responding to sexual and gender‐based violence against women and children in low‐ and middle‐income countries is still relatively low, and almost non‐existent with respect to children. However, with increasing interventions, future high quality studies appear to be increasing.

### 1.2 What is this EGM about?

The problem of sexual and gender‐based violence (SGBV) against women and children is prevalent in low‐ and middle‐income countries (LMIC). Adoption of ICT has increased exponentially throughout the world in the past decade. As new types of ICT interventions are being implemented with the purpose of preventing and/or responding to SGBV against women and children in LMICs, donors and policymakers have been looking for evidence based on rigorous studies that assess the effectiveness of such interventions. This EGM shows the available evidence from individual studies and systematic reviews.

**What is the aim of this EGM?**

The primary goal of this EGM is to establish a baseline for the state of evidence relating to the use of ICT for preventing and responding to sexual and gender‐based violence against women and children in low‐ and middle‐income countries.

### 1.3 What studies are included?

The EGM includes systematic reviews, academic studies and gray literature from January 2005 to July 2021, focusing on:

1. women and girls
2. all children, aged 18 or under; and
3. SGBV prevention and/or response service providers in LMIC.

Ten studies met the inclusion criteria. Of these, two are systematic reviews, two are scoping reviews and the remaining six are individual studies (two of which had separately published protocols, which are also included in the EGM).

Studies and reviews were categorized by type of intervention, and outcomes reflected the RESPECT Women Framework for preventing violence against women (World Health Organization, 2019a) and the INSPIRE Seven Strategies for Ending Violence against Children (World Health Organization, 2016).

### 1.4 What are the main findings of this EGM?

No studies focused on using ICT exclusively for children. There is some evidence of impact in the areas of using ICT:

1. to influence gender norms related to SGBV prevention and/or response;
2. in the form of mobile applications to increase safety and security; and
3. to collect evidence related to SGBV incidents and user perceptions of SGBV services.

### 1.5 What do the findings of the EGM mean?

The results indicate a need for more rigorous studies on using ICT for SGBV prevention and/or response conducted in LMIC.

Specific research gap areas include: using harmonized and standardized impact indicators, child‐focused interventions, and studies explicitly linking how ICT contributes to intermediate outcomes related to SGBV (identified under the RESPECT and INSPIRE frameworks) and ultimately to prevention and response outcomes.

### 1.6 How up‐to‐date is this EGM?

The authors searched for studies published up to July 2021.

## 2 BACKGROUND

### 2.1 Introduction

#### 2.1.1 The problem, condition, or issue

##### Global sexual and gender‐based violence trends

The problem of sexual and gender‐based violence (SGBV) against women and children (regardless of gender) is widespread globally, and particularly prevalent in low‐ and middle‐ income countries (LMIC) (those listed by the Organization of Economic Co‐operation and Development as “low” and “lower‐middle” income) stresses from socioeconomic and political pressures tend to be more exacerbated. Further, prevalence of violence against all women (whether in the form of intimate partner violence (IPV), or perpetrated by non‐partners), tends to be higher in LMIC (World Health Organization, 2013). Despite higher prevalence, there is relatively less investment in SGBV research in LMIC compared to HIC (Coll et al., 2020).

SGBV^1^ is a ubiquitous global phenomenon. It occurs in many forms and contexts including IPV (physical, sexual, and emotional abuse); sexual violence; conflict‐related sexual violence; forced and early marriage; trafficking; female genital mutilation; femicide; homophobia and transphobia; and sexual harassment and abuse. What unites these diverse manifestations of violence is that, at their core, they are violent acts associated with the socially assigned gender differences between males and females, often exploiting individual and social power imbalances.

Having complete accurate and timely data on the SGBV is still more of a goal than a reality. Available country data indicates that between 15% and 76% of women are targeted for physical and/or sexual violence in their lifetime (World Health Organization, 2013). 38% of murders of women globally are committed by their intimate partners (World Health Organization, 2021).

Globally, up to 50% of sexual assaults are committed against girls under 16 years old (World Health Organization, 2013). Global data estimates that in 2002, 150 million girls under the age of 18 experienced some form of sexual violence (UN Women, 2019). Femicide, or the intentional killing of women based on their gender, has increased globally since 2012 by roughly 80% (United Nations Office on Drugs and Crime, 2019) with Africa and Latin America having the highest rates per 100,000 female inhabitants (3.1 and 1.6 respectively) (United Nations Office on Drugs and Crime, 2019). Individual countries, such as El Salvador (6.8 per 100k females) and Honduras (5.1 per 100k females), have even higher rates (UN Gender Equality Observatory, 2020).

The 2020 Covid‐19 pandemic further exacerbated acts of SGBV with some country reports of IPV increasing along with the government‐mandated lock‐downs and the stress from the loss of livelihoods and confinement (Mlambo‐Ngcuka, 2020). It is anticipated that COVID‐19 will disrupt efforts to end child marriage, potentially adding 10 million child marriages in the next decade that could have been averted to the 650 million women alive today who were married as children (UNICEF, 2021). For every 3 months of COVID‐19 lockdowns, an additional 15 million cases of SGBV are expected (UNPF, 2020).

##### Information and communication technology

Information and communication technology (ICT), also referred to as “digital technology,” specifically the use of mobile phones, tablets, and web‐based communications (laptops) to address multiple development challenges in low‐ and middle‐income countries, has increased exponentially in the past decade (UNESCO, 2020; World Bank Group, 2016). This trend towards ICT uptake is especially true of young people with an average of 83% of those aged 18–29 owning a mobile phone (Ippoliti & L'Engle, 2017 citing Pew Research Center, 2014). Evidence, supported by methodologically rigorous research, of the impact of using ICT in areas such as health, has indicated that *if used properly*, ICT can increase the impact of interventions and address gaps and challenges inherent with the delivery of interventions (World Bank Group, 2016). The World Bank estimates that the number of internet users tripled from 1 billion in 2005, to 3.2 billion at the end of 2015, and that “70% of the bottom one‐fifth of the population own a mobile phone” (World Bank Group, 2018).

The use of ICT (mobile phone reminders) has been proven as an effective strategy to ensure that patients adhere to their appointed antiretroviral regimens (Lester et al., 2010; Mills & Lester, 2019). The World Health Organisation recognized the need for an evidence base to support ICT use increase in health areas such as maternal, newborn, and child health and HIV and AIDS, and in 2019, published a Guideline of recommendations on digital interventions, supported by a critical evaluation of evidence (World Bank Group, 2018). The Guideline, “identifies evidence gaps to inform member states and streamline includes future research investments” supported by contributions from eleven Cochrane reviews (Cochrane Collaboration, 2019).

Stakeholders working in the area of SGBV, such as the Sexual Violence Research Initiative (SVRI) and the World Bank, have recognized and acknowledged the increased use of ICT to both prevent and respond to SGBV globally (Hayes, 2014; Sexual Violence Research Initiative, 2017). As in many health areas, ICT is being used as a tool to facilitate interventions that are known to be effective in addressing SBGV (for prevention and response) outlined in globally accepted evidence‐based frameworks for preventing and addressing violence (including, but not limited to, SGBV) such as the RESPECT Preventing Violence against Women Framework (World Health Organization, 2019a) and the INSPIRE: Seven Strategies for Preventing Violence (violence against children) (World Health Organization, 2016).

Published studies on the use of ICT directly for SGBV prevention and/or response, notably in LMIC, are scarce. Other than several recent but narrow systematic reviews that (1) provide an initial analysis and functional categorization of mobile phone applications addressing violence against women (Eisenhut et al., 2020); (2) examine web‐ and mobile‐based delivery methods of IPV prevention (Anderson et al., 2019); (3) examine the effect of eHealth interventions compared with standard care on reducing IPV, depression, and posttraumatic stress disorder (PTSD) among women exposed to IPV (Linde et al., 2020); and (4) identify the effectiveness of ICT‐based IPV interventions (El Morr & Layal, 2020), a dearth of available peer‐reviewed published research exists. The majority of those studies that are published and readily available took place in higher income countries. There have been few attempts to identify and systematically review the research and evidence of outcomes and impact attributable to using ICT *specifically for* SGBV prevention and/or response in LMIC.

While evidence has been emerging examining the gender implications connected with the use of ICT including various benefits to women and girls (as well as children in general), some studies and literature have also raised questions about the role of ICT in exacerbating or contributing to SGBV (Crabtree & Geara, 2018).

### 2.2 The intervention

#### 2.2.1 Describe the intervention(s)

##### What is the scope of ICT interventions for SGBV?

ICT for SGBV prevention and/or response interventions involves a broad scope given the complex nature of SGBV in general. Further, the ICT component of an intervention, is generally not the SGBV prevention and/or response intervention *per se*, but its method of delivery to the end user (i.e., IPV clinical screening tools that are tablet rather than paper‐based). Modes of ICT include mobile phones, tablets, and web‐based applications using laptop computers. We included ICT interventions for the prevention of SGBV against women and children, as well as for responding to SGBV by improving survivors' access to services and preventing the re‐occurrence of SGBV. We excluded prevention and response interventions addressing violence that are not related to socially ascribed differences between males and females. For example, we excluded literature discussing violence connected to the disciplining of children.

The scope also includes using ICT to *achieve intermediate outcomes* that are part of causal pathways for (1) preventing SGBV against women and children in low‐ and middle‐income countries; and (2) responding to SGBV by improving survivors' access to services. These intermediate outcomes included those connected with evidence‐based interventions contributing to the prevention of violence under the RESPECT and INSPIRE frameworks.

We *excluded* the uses of ICT if they are not specifically and purposefully used to deliver or fill gaps in prevention and response interventions for SGBV.

We attempted to identify ICT supported interventions that have as an objective either (1) SGBV prevention; and/or SGBV response (improved access for SGBV survivors to services); or (2) an intermediate outcome that is part of the evidence‐based causal pathway to either SGBV prevention or improved access for SGBV survivors to services (with reference to the RESPECT and INSPIRE frameworks). These intermediate outcome areas are connected with root causes of SGBV established through global evidence. We attempted to identify only studies related to interventions if they are delivered using ICT and describe the role of ICT in facilitating the delivery of the intervention.

#### 2.2.2 Examples

Illustrative examples of prevention interventions using ICT include gaming applications or “apps” which contain messaging aimed to sensitize and change gender norms, attitudes, and behavior of male and female adolescents to the negative consequences of gender bullying and violence; web‐based applications alerting friends and contacts to intervene if a woman feels threatened on a date; and web‐based maps indicating the geographic locations where incidents of SGBV have occurred.

#### 2.2.3 Who delivers the intervention?

SGBV prevention and response interventions are typically delivered by civil society groups, non‐governmental organizations (NGOs), governments, or a collaboration that may also include universities. A separate technology partner, unless the group, NGO or government has internal technological capacity, provides the technological input, coding the ICT tool whether it is a mobile phone application and/or web‐based laptop computer application, conducting a feasibility assessment (examining the conditions such as connectivity, usability, etc.), and delivering necessary training to the targeted users of the technology. The technologists work (or are supposed to work) hand‐in‐hand with the program specialists (e.g., child protection, gender, and SGBV specialists) to ensure the interventions supported by technology are appropriately designed, and most importantly, deployed in accordance with the “do no harm principle.”

SGBV interventions may also be delivered in the form of applications (software) that can be downloaded onto phones, tablets, and computers (e.g., “laptops”), and used without additional involvement of external parties.

#### 2.2.4 Who are the targeted groups?

For the purpose of this EGM, the primary targeted groups are:

- Women and girls in LMIC
- Boys in LMIC (vulnerable to, and as survivors of SGBV)
- Children and others who are vulnerable to SGBV, including transgender and gender‐nonconforming persons, whether or not they identify as female.

We define “children” based upon the definition under the Convention on the Rights of the Child, to “include male and females being below the age of 18 years unless under the law applicable to the child, majority is attained earlier” (United Nations, 1989).

However, we also included interventions that target intermediary groups who are part of the causal pathway to preventing SGBV against women and children and increase access to SGBV services for survivors including preventing the recurrence of SGBV:

- Potential perpetrators (i.e., men and boys) in LMIC
- Intimate partners in LMIC
- Service workers (e.g., health, social, police) in LMIC
- Organizations working in the area of SGBV or in any of the outcome areas relating to the prevention and response of SGBV identified under the RESPECT and INSPIRE frameworks
- Communities and community leaders in LMIC

#### 2.2.5 Prevention/response

In addition to conceptualizing interventions as intermediate or primary in terms of their targeted user, we also considered whether the outcomes are for prevention or response using the following framework:

- *Primary prevention*: Aimed at the whole community or at men and boys specifically to stop SGBV before it occurs. Addresses root causes of violence (White Ribbon Australia, 2016). Examples include men's engagement work and SGBV awareness initiatives, in general. A community may also include institutions as well as populations, such as school systems, healthcare networks, peacekeeping forces, etc.
- *Secondary Prevention*: Focuses on preventing violence from continuing or escalating. Aimed at individuals and groups at risk of being exposed, or at perpetrators of violence. May include home visits from social workers to household members who are at risk of violence; or behavioral change programmes for men with anger management problems (White Ribbon Australia, 2016). At the institutional level, secondary prevention encompasses “risky environments,” for example, schools that have incidences of SGBV amongst girls, localities where sex work is common and SGBV has been reported (especially amongst those who engage in survival sex work), forced migration response camps, etc.
- *Tertiary Prevention* (*includes response*): Aimed at survivors and perpetrators of SGBV. Implemented after the violence has occurred and focuses on minimizing the impact of violence, restoring health and safety, and preventing violence from occurring again (Carmody et al., 2014; White Ribbon Australia, 2016). Ideally, SBGV response interventions include a tertiary prevention component. We note those response interventions which do.

### 2.3 Why it is important to develop the EGM

SGBV is a persistent global phenomenon. ICT represents a potential facilitator for SGBV prevention and/or response. Very little evidence‐based knowledge exists regarding its use, effectiveness, and feasibility in LMIC. This scarcity of evidence impedes important government, NGO, and activist efforts to better understand ICT's potential to contribute to preventing and responding to SGBV. An EGM is a tool for better understanding this evidence landscape including: description of where ICT work is being done (context); focus population; whether it is to prevent or respond to SGBV; and the quality and the diversity of the body of evidence that exists. An EGM serves to establish a baseline to inform future research efforts.

Systematic reviews that address the evidence related to preventing SGBV and the use of ICT to achieve other health outcomes (e.g., relating to HIV, maternal health, etc.) have been published. However, there are few to no reviews (systematic or EGMs) identifying evidence related to the use of ICT for outcomes related to prevention and/or response to SGBV against women and children in LMIC. Those that exist are limited in scope and focus on higher‐income countries (Anderson et al., 2019). (See Section 4.1 “EGMs: Definition and Scope”).

Further, as the use of ICT expands at such a rapid pace both within and outside the context of SGBV prevention and/or response, the need for methodologically rigorous research requires advocacy amongst both the ICT and SBGV research communities. We confirmed that few rigorous studies exist meeting the eligibility criteria, but we also believe that determining a baseline of existing evidence, as few as they may be, is an essential starting point for an advocacy strategy encouraging more rigorous studies to be conducted.

To further underscore the timeliness and importance of this review, we note that emerging reports show a correlation between the Covid‐19 pandemic and increasing gender‐based violence, especially IPV (Chukwueke, 2020; Mlambo‐Ngcuka, 2020). Accordingly, we attempted to explore emerging evidence related to SGBV during the pandemic, and the role ICT can play in prevention and/or response interventions. For example, the Gender‐Based Violence Information Management System in Mali generated data demonstrating a 35% increase in SGBV between April 2019 and April 2020 (UNFPA, 2020). This SBGV trend has also been noted in other African countries. At the time of submission of the EGM, the authors did not identify eligible studies exploring this important intersection.

An EGM that locates evidence connected to the operationalization and implementation of ICT for preventing and responding to SGBV is not only warranted, but critical for implementers to comply with the principle of “do no harm” when delivering interventions in potentially vulnerable contexts.

## 3 OBJECTIVES

*The goal of this EGM is to establish a baseline for future research of the evidence connected with using ICT for the prevention of, and response to, SGBV against women and children in LMIC. As part of establishing the baseline, the primary objectives of this EGM are to*:

(1) identify evidence of effectiveness for the use of ICT targeting the prevention of, and response to, SGBV against women and children in LMIC;
(2) identify key gaps in the available ICT and SGBV prevention and/or response evidence;
(3) identify research methodology issues reflected in the current evidence;
(4) identify any clusters of evidence in one or more ICT interventions suitable for systematic review;
(5) identify enabling factors associated with effective interventions using ICT for the prevention of, and response to, SGBV against women and children in LMIC; and
(6) provide a structured and accessible guide to stakeholders for future investment into interventions and research using ICT for SGBV prevention and response in LMIC.

Types of evidence considered: methodologically rigorous studies evaluating effectiveness and implementation processes, including experimental, quasi‐experimental, and qualitative studies, systematic reviews, and certain gray literature.

## 4 METHODS

### 4.1 EGM: Definition and scope

EGMs represent a relatively new approach to identifying and classifying available research and evidence for a broad scope or topic, such as ICT for SGBV prevention and/or response. EGMs offer a rigorous and systematic process to “map” what evidence exists and where also gaps in evidence exist. They are ideal for topics where there is little understanding of the research landscape; or those that incorporate several potential interventions with multiple outcomes and may lack widely available studies. EGMs differ from systematic reviews in that they can identify clusters of evidence that may be further analyzed by meta‐analysis with each cluster representing a potential systematic review in itself (Saran & White, 2018).

An EGM is ideal for identifying the evidence connected to the use of ICT for the prevention and response of SGBV against women and children in LMIC (those listed by the Organization of Economic Co‐operation and Development as “low” and “lower‐middle” income) precisely because the scope of SGBV and ICT encompasses several potential intervention designs with multiple target populations and outcomes. An EGM offers a systematic approach to locate and analyze what evidence exists for using ICT to prevent and respond to SGBV and highlight evidence gaps. While EGMs do not include meta‐analysis per se—the clusters they can identify are often readily developed into individual systematic reviews. In search strategy and methodological rigor, however, EGMs differ little from systematic reviews.

Systematic reviews require precision with inclusion and exclusion criteria to properly meta‐synthesize data. Given that we expected little, but diverse available evidence for SGBV and ICT, a systematic review was deemed likely to include very few studies and miss other key evidence that is necessary to understand the broader context of ICT for SGBV prevention and/or response research in LMIC. An initial EGM, in contrast, allows us to first identify what, if any, evidence clusters do exist, and further develop these into individual reviews, if possible.

### 4.2 Stakeholder engagement

A steering committee of SGBV and ICT experts, primarily including representatives from LMIC, have advised and assisted in the conceptualization of the EGM and contributed to a concurrent landscape review that was conducted in conjunction with the EGM. The committee included experts in ICT, gender and SGBV, and were comprised mostly of women. The committee included academics, civil society actors, and activists. The committee assisted in the identification of stakeholders for key informant interviews, interventions, and approaches that helped guide the EGM. The landscape review helped to identify what types and where ICT for SGBV prevention and/or response interventions are being or have been implemented in LMIC, but not reported in peer review literature. In July 2021, an online participatory workshop was convened, including the steering committee, global specialists, academics, implementers, and donors who work with ICT and SGBV, to review preliminary findings of the landscape review and EGM to develop a framework in the form of an ecological model for analyzing the use of ICT to prevent and respond to SGBV in LMIC (Mechael et al., 2021).

### 4.3 Conceptual framework development and scope

The framework for this EGM was developed through consultation with stakeholders (key informant interviews), a global steering committee of experts, and through the application of existing violence prevention frameworks (e.g., RESPECT and INSPIRE), theories and policies. As a complement to the EGM, a landscape review of interventions using ICT to prevent and respond to SGBV against women and children in LMIC captures work being conducted by activists, civil society, NGOs, and other groups that are not represented or published in traditional academic and research settings (and therefore likely to not be identified through systematic searches of peer reviewed literature).

**Figure 1:**
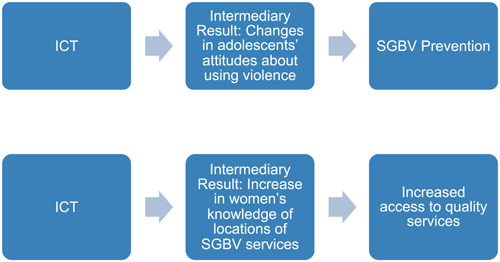
Intermediate outcomes and causation

Because of challenges measuring primary SGBV outcomes, especially prevention, different groups may be targeted as mediators (intermediaries) for achieving intermediate outcomes that contribute to, or are inferred, based upon available evidence to contribute to, SGBV prevention and response primary outcomes. The research team acknowledges that interventions, for the purpose of ultimately preventing and/or responding to violence, may have the objective of achieving an *intermediate outcome* that contributes to the primary outcome (or impact) of either preventing SGBV or responding to SGBV (e.g., facilitating access to services, including the prevention of the SGBV recurrence for survivors of SGBV). Often there is more than one intermediate outcome, attributable to multiple interventions, which together form a causal pathway leading to the primary outcome (or impact) such as SGBV prevention (e.g., women's economic empowerment and strengthening relationship/life skills) (World Health Organization, 2019a) (see Figure 1).

We attempted in our searches to identify studies that evaluated intermediate results that may be components of Theories of Change or are components of an ecological model that contribute to, or are prerequisites leading to primary outcomes (or impact), specifically prevention and response. It is difficult to measure primary prevention outcomes or impact (e.g., a reduction in SGBV prevalence) without a longitudinal study.

Established evidence related to the prevention of sexual violence supports inferences that achieving one or more intermediate outcomes will necessarily lead to, or contribute to prevention (reduced SGBV prevalence), or effective response interventions (greater access to services). It was anticipated and acknowledged that studies may tend to more likely evaluate the effectiveness of an intervention in achieving an intermediate outcome related to the prevention or response to SGBV against women and children, referring to the RESPECT and INSPIRE frameworks as guides (assuming that evidence regarding the prevention of violence in general can apply to the prevention of the more specific SGBV). Based upon evidence, we sought to infer that the intermediate outcome will either directly lead to, or is a necessary ingredient for achieving the primary outcome/impact (e.g., SGBV prevention or improved access to quality SGBV services).

We also attempted to search for studies that targeted and measured outcomes connected with groups other than women and children. These targeted intermediary groups include SGBV service providers (social workers, health providers, prosecutors, etc.), potential and actual SGBV perpetrators (including intimate partners), community members, and organizations.

**Figure 2:**
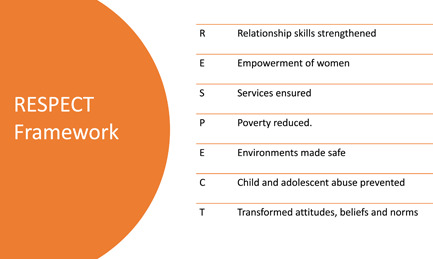
RESPECT Preventing Violence Against Women Framework (WHO, 2019)

**Figure 3:**
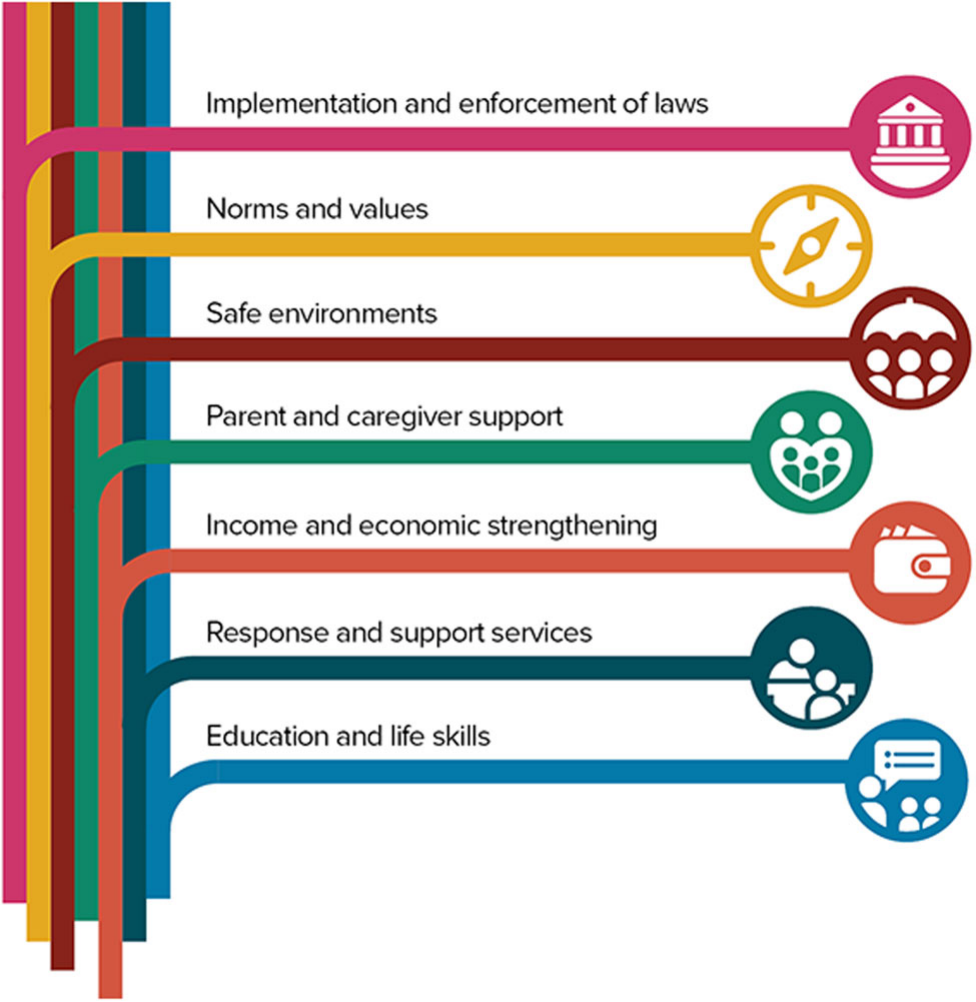
INSPIRE Seven Strategies for Preventing Violence against Children (WHO, 2016)

The World Health Organization's RESPECT evidence‐based framework for the prevention of violence against women (including SGBV) is useful to conceptualize SGBV intervention outcomes (World Health Organization, 2019a). The RESPECT framework conceptualizes risk and protective factors for violence (including SGBV) across four environmental levels: individual, interpersonal, community, and society. These divisions offer an important roadmap to consider SGBV outcomes that go beyond individual attitudes, knowledge, and behaviors and look towards structural outcomes at both the community and society level. Similarly, the INSPIRE Seven Strategies for Ending Violence Against Children (World Health Organization, 2016) presents evidence‐based strategies for preventing and addressing violence, including SGBV, against children. The RESPECT and INSPIRE frameworks have been used to inform the EGM's outcome dimensions (see Figures 2 and 3).

We also drew heavily from the results of an online workshop we held with SGBV and ICT stakeholders held in August 2021.

**Figure 4:**
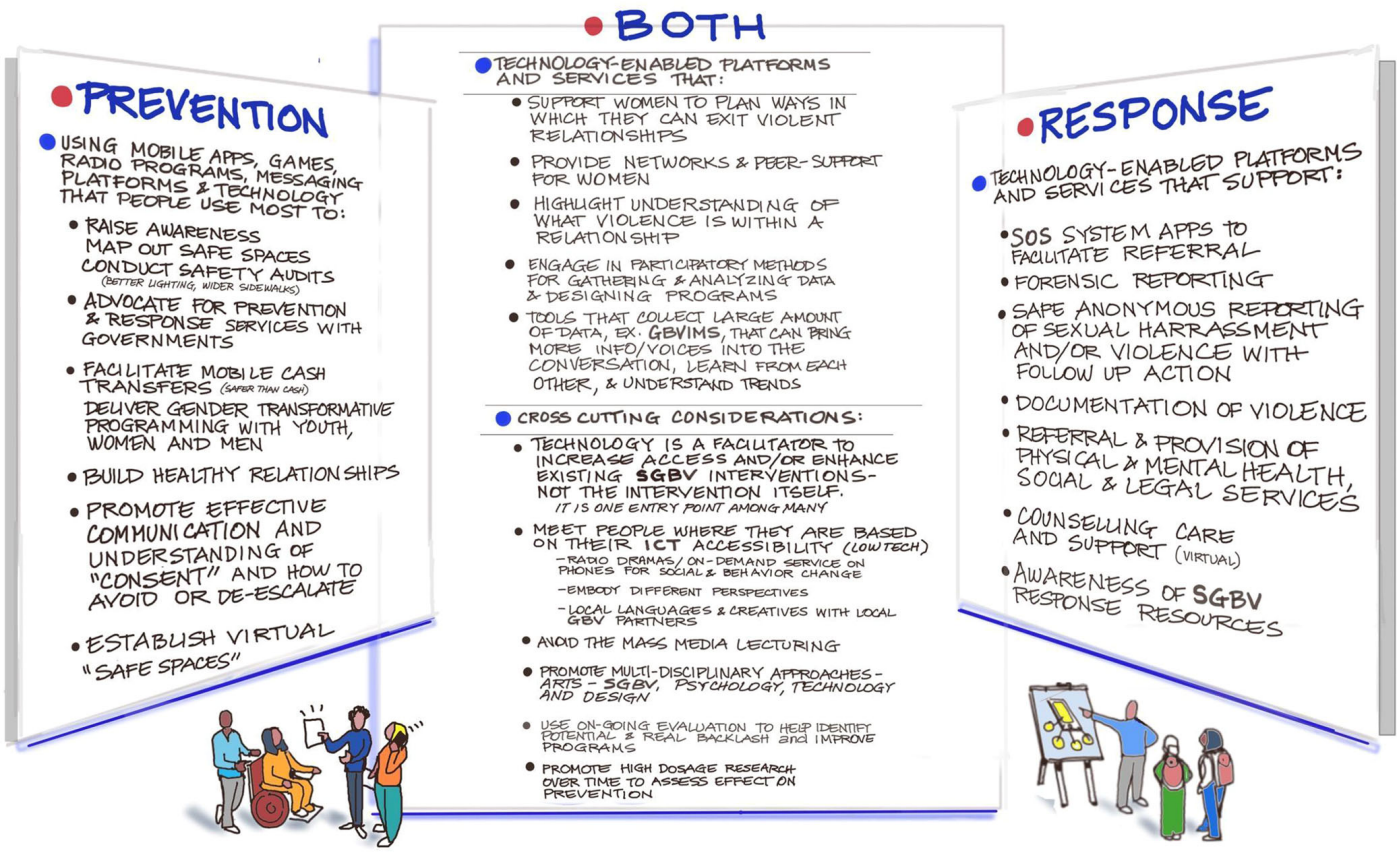
Conceptual framework for ICT interventions for SGBV (developed at Stakeholder Workshop).

**Figure 5:**
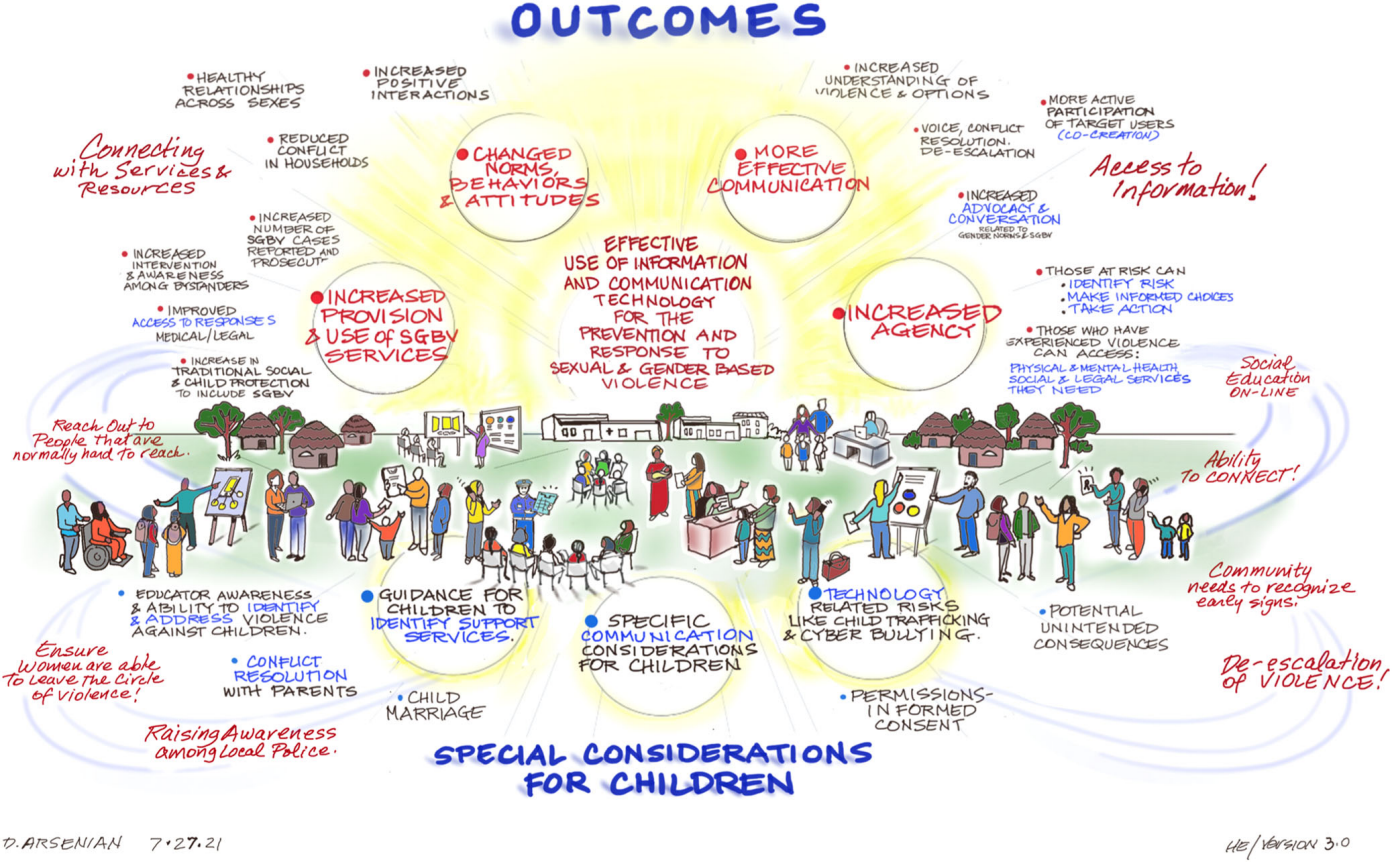
ICT SGBV outcomes identified in Stakeholder Workshop.

**Figure 6:**
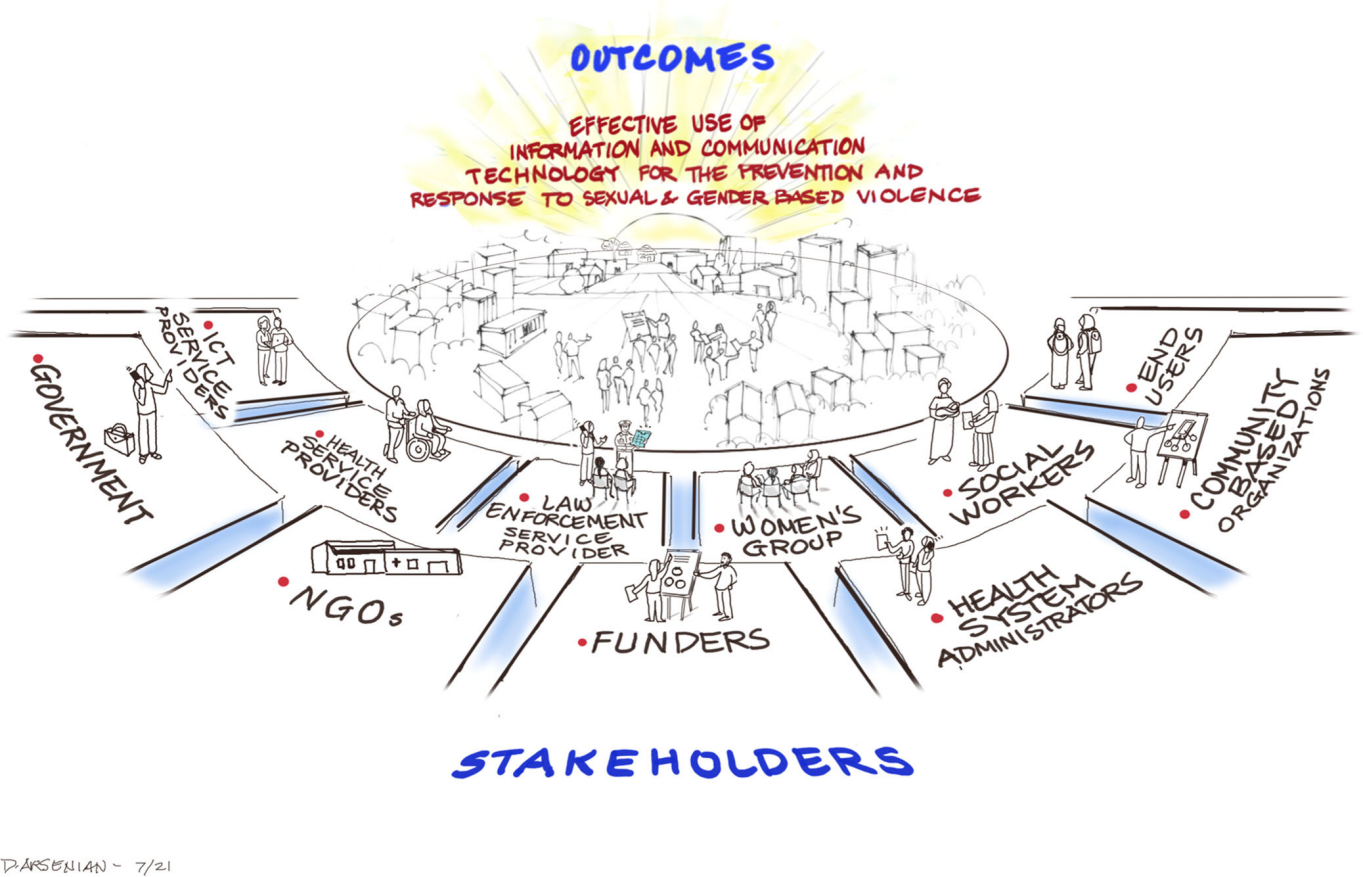
Stakeholders connected with using ICT for SGBV prevention and response identified in Stakeholder Workshop.

See Figures 4, 5, 6 for the Stakeholder Workshop Identification of ICT Interventions and Outcomes for Preventing and Responding to SGBV.

### 4.4 Dimensions

The conceptual dimensions used for coding the EGM reflect key elements of using ICT for the prevention of, and response to, SGBV for intervention design, organized by *type or description of ICT intervention, target population (or user) intervention outcome, study design*, and other descriptive factors such as *region* and *sponsor organization*.

We organized the EGM data along the axes of *intervention context* by *intervention outcome*. The columns represent the context of the ICT interventions (IPV focus, focus on prevention and/or response, discussions of sustainability, and unintended consequences). While the rows lay out different intervention outcomes. We developed the codes for our outcomes by using the WHO RESPECT Framework (World Health Organization, 2019a). Our outcomes include both primary (SGBV incidence/prevalence) and intermediary outcomes, such as women's empowerment and access to SGBV‐related services. We also coded for studies that were descriptive in nature and did not include explicit outcomes, as well as coded for feasibility studies which evaluated a potential or developing intervention. Included studies that had more than one outcome were coded more than once as appropriate. However, we attempted to avoid the overuse of double coding.

We segmented our EGM dimensions by study design (RCT, Qualitative, Quantitative, or Mixed‐Methods). There was no double‐coding for study design. We included many other descriptive filters such target population and type of ICT device used by the intervention.

#### 4.4.1 Types of study design

Given that we expected to find few published studies related to ICT for SGBV prevention and/or response we included as many study designs as possible, including those that are ongoing. As such, we included:

- Systematic reviews of experimental or quasi‐experimental studies so long as at least one of the studies included was conducted in a LMIC. We used a broad scope for inclusion under “systematic reviews” as we anticipated few reviews, if any, would turn up in the search. Accordingly, reviews that may be more accurately deemed “scoping” or landscape reviews which do not reflect the same level of rigor as systematic reviews that may be published by Campbell or Cochrane for example, may have not been automatically excluded.
- Experimental (e.g., randomized control trials and other experiments with random assignment, including QRCTs—quasi‐randomized controlled trials where participants are allocated by, for example, alphabetically, without revealing personal identifiable information; NRCTs—non‐randomized controlled trials where participants are allocated by other actions controlled by the researcher; non‐randomized studies, where allocation is not controlled by the researcher and two or more groups of participants are compared).
- Quasi‐experimental with well‐defined comparison group, including a well‐defined comparison group, non‐randomized controlled trials, cohort studies, case‐control studies, and cross‐sectional analytical studies.
- Rigorously designed qualitative research guided by precise and clear research questions; with adequate data collection to address research questions; interpretation of results substantiated by data, and coherence between qualitative data sources, collection, analysis and interpretation.

We also determined to include studies without comparisons of cohorts if they examine elements of ICT implementation useful to SGBV and ICT stakeholders, especially those that examine cost‐effectiveness, feasibility of implementation, or acceptability of a given intervention

#### 4.4.2 Types of intervention/problem

The use of ICT is not necessarily the intervention itself, but a means to *support and enable* the underlying intervention (e.g., tool for communicating messages connected with changing social norms for SGBV prevention), or to address obstacles and challenges in the delivery of an intervention (e.g., communicating essential information to hard‐to‐reach populations regarding locations of SGBV response services). Accordingly, we categorized interventions by type of ICT application. We identified ICT interventions according to common types of ICT applications, adapting, in part, frameworks developed in the mHealth field, such as the mHealth and ICT Framework for Reproductive, Maternal Newborn and Child Health (RMNCH) (Labrique et al., 2013).

Ideally, we sought to identify studies that reported on an ICT intervention being compared to an existing non‐ICT‐based intervention—for example, tablet‐based screening tools compared to the same tool in paper form, or web‐based workshops on gender attitudes compared to in‐person workshops.

Examples of types of ICT applications (some of which may overlap) include:

(1) Gender norms, attitude, and behavior change communication and education
(2) Data collection and reporting, including the assessment of services
(3) Electronic decision support or “safety applications” (information, protocols, algorithms, checklists)
(4) Messaging
(5) Provider‐to‐provider communication
(6) Provider training and education
(7) Mapping (of services, incidents of violence, etc.)
(8) Gaming
(9) Hotlines
(10) Referrals

See Supporting Information: Appendices for Table of Illustrative Interventions

#### 4.4.3 Types of population (as applicable)

We included studies that focused on two types of populations living in LMIC:

- *Primary* or those vulnerable or survivors of SGBV: women and children, especially girls, those vulnerable to other forms of SGBV (ex. transphobic violence).
- *Intermediaries* or those intermediate in the SGBV prevention and/or response causal pathway: potential perpetrators, perpetrators, first responders, and any others intermediary agents (community health workers, social workers, organizations, etc.).

#### 4.4.4 Types of outcome measures (as applicable)

We attempted to identify ICT supported interventions that have as an outcome either (1) the primary outcome/impact of SGBV prevention and/or SGBV response (improved access for SGBV survivors to services); or (2) an intermediate outcome that is part of the causal pathway to either SGBV prevention, or improved access for SGBV survivors to services as identified under the RESPECT and INSPIRE frameworks.

With regard to studies with primary outcomes/impact, we did not anticipate identifying many studies that measured changes in SGBV prevalence. We anticipated that we would be more likely to identify studies that measured changes in the uptake of SGBV‐related response services.

The intermediate outcomes may reflect any of the evidence‐based strategies for preventing SGBV reflected in the RESPECT and INSPIRE frameworks such as transformed knowledge, behaviors, beliefs and norms, environments made safe, social or economic empowerment of women, reductions in poverty, relationship skills strengthened (World Health Organization, 2016, 2019a), or multiple intermediate outcomes.

We searched for intermediate outcomes when there is an explicit connection with SGBV prevention and/or response. For example, we excluded poverty reduction interventions (a RESPECT strategy) that do not have an explicit SGBV connection. We excluded interventions that create safe environments (another RESPECT strategy) without a specific intent to prevent SGBV.

These intermediate outcomes may be related to SGBV prevention and/or response. Examples include:

- Changes in knowledge and awareness by those vulnerable to SGBV for how to avoid and prevent SGBV. Example: *a mobile phone app that helps women recognize risk factors for IPV*.
- Changes in knowledge and awareness by SGBV survivors about where and how to access SGBV services. Example: *a web‐based format that allows SGBV survivors to report SGBV incidents to police from their mobile phones and directs to local medical services for first‐response*.
- Changes in social norms, attitudes, and behaviors among men and boys, and intimate partners regarding SGBV, which ultimately should lead to reducing incidents of IPV. Example: *a gaming app that helps adolescent boys develop more gender‐equitable attitudes and behaviors*.
- Changes in community social norms, attitudes, and behaviors towards SGBV. Example: *a social media campaign targeting community attitudes or beliefs regarding common SGBV myths (e.g., women should acquiesce to violence perpetrated by their partners), which ultimately should lead to less acceptability and therefore a reduction of SGBV in the community*.
- Service providers building improved capacity and capabilities (knowledge) in providing SGBV services to SGBV survivors. Example: *mobile phone‐based tools to assist social workers in better identifying SGBV risk factors or signs of SGBV when making home visits, which ultimately should lead to a reduction of SGBV, increased access to services and/or reduce the re‐occurrence of SGBV*.
- Changes in the economic empowerment of women and family household incomes and improved relationship skills between intimate partners. Example: *a mobile phone application that guides women in women‐led village savings and loan associations and provides relationship building curriculum for discussion which should contribute to the reduction of IPV*.

- Reductions in alcohol intake: Example: mobile apps and other ICT tools promoting the reduction of alcohol intake, a key contributor to SGBV.

#### 4.4.5 Other eligibility criteria

We focused on interventions implemented in LMIC, but took note of interventions implemented in higher income countries (HIC) when useful to provide context, and for comparison in the descriptive narrative and analysis.

##### Types of location/situation (as applicable)

Eligible studies were carried out in LMIC. If we were considering systematic reviews, at least one of the studies covered under the systematic review had to be carried out in a LMIC.

##### Types of settings (as applicable)

We included ICT interventions that have home‐based individual end‐user settings (mobile‐phone, tablet, or other web‐based settings); as well as those that may encompass clinical settings (e.g., ICT‐based SGBV screening tools). We included only interventions in LMIC. Interventions in HIC have been excluded from the EGM, but we refer to interventions in HIC when useful to provide context in the descriptive narrative and analysis.

The following settings and contexts have been included, but not necessarily limited to:

- Humanitarian and emergency settings (including refugees and internally displaced persons or “IDPs”)
·Domestic settings (IPV)
- Children in the armed forces
- Trafficking
- Schools
- Conflicts, war, fragile states (post‐conflict)
- Mobile children and women (including migrants)

#### 4.4.6 Search methods and sources

The full original protocol for this EGM was previously published by the Campbell Collaborations. (Philbrick et al., 2021).

##### Electronic searches

Relevant studies have been identified through electronic searches of bibliographic databases, research networks, government policy databanks, and internet search engines. The searches included studies published from 2005 and forward (The search dates are restricted as the results of the use of ICT for health interventions were relatively rare prior to 2005). Results in English, Portuguese and Spanish (languages spoken by the reviewers) were reviewed. The bibliographies of relevant reviews and included studies were used to identify additional references for review. We conducted forward citation searching using the website Google Scholar.

Any changes in eligibility criteria were agreed prospectively between the members of the review team. These have been documented and reported as a discrepancy from protocol in the manuscript.

Search terms were developed based on terminology representative of gender and ICT implementation and dissemination research and included search filters used in previous reviews. We particularly leveraged globally accepted terminology related to SGBV in globally accepted frameworks and guidance including INSPIRE: Seven Strategies for Ending Violence Against Children (World Health Organization, 2016), RESPECT Women: Preventing Violence Against Women (World Health Organization, 2019a), Essential Services Package for Women and Girls Subject to Violence, Sexual Violence Researcher Initiative, Together for Girls, and the Violence Against Women Working Group of the International Federation of Obstetrician‐Gynecologists (FIGO). We aligned with the body of studies incorporating ICT‐related terminology, such as the use of “mHealth” and “eHealth” that have been standardized over the past 10 years. The search strategy for PubMed is presented under “Sample Search Terms” below and was adapted for the other databases.

##### Gray literature

We reviewed relevant gray literature, developing a separate search strategy including all of the following elements:

1. *Searches of gray literature databases* such as ProQuest Dissertations and Theses, etc.
2. *Targeted Google searches* that were based on the search terms used with electronic databases
3. The *targeted screening of relevant sector and implementation websites (Landscape Review)*, including content from civil society, feminist groups NGOs, and other activist web‐based content relevant to the prevention and response of SGBV in LMIC.

As ICT for SGBV prevention and/or response interventions span multiple disciplines and contexts, social science, public health, education, humanitarian, and ICT‐related electronic databases were searched. Search terms varied by database but generally included three blocks of terms and appropriate Boolean or proximity operators, if allowed. Blocks included terms that addressed (1) intervention; (2) population; and (3) outcomes.

##### Bibliographic databases searched

- Association for Computing Machinery (ACM) Digital Library
- African Journals Online (AJOL)
- Business Source Complete
- Campbell Collaboration Library
- ClinicalTrial.gov
- Cochrane Library
- Education Resources Information Center (ERIC, via ProQuest)
- EPPI‐Centre Systematic Reviews
- Google Scholar
- IMISCOE
- LILACS (Latin American and Caribbean Health Sciences Literature)
- MEDLINE
- Mendeley
- ProQuest (all databases)
- PsycARTICLES
- PubMed

##### Searching other resources

Websites and other internet repositories searched included:

- ScienceOpen
- Sexual Violence Research Initiative (SVRI)
- Social Science Research Network (SSRN)
- SpringerLink

Copies of relevant documents from Internet‐based sources were made. We recorded the exact URL and date of access.

*Snowballing*: Reference lists of included studies and relevant reviews were searched for potential new literature.

*Personal contacts*: Personal contacts with national and international researchers were considered to identify unpublished reports and ongoing studies.

##### Sample search terms (not exhaustive)

(ICT [tiab] OR Information*‐ communication*‐ technolog* [tiab] OR tablet_ OR mobile‐phone [tiab] OR web [tiab] OR cell‐phone [tiab] OR electronic [tiab] OR digital [tiab] OR gam* [tiab]) OR podcast (tiab)) AND (“Sexual gender based violence” [tiab] OR “gender‐based violence” [tiab] OR rape [tiab] OR SGBV [tiab] OR GBV [tiab] OR intimate‐partner‐violence [tiab] OR IPV [tiab] OR FGM OR female‐genital‐mutilation [tiab] OR violence‐against‐children [tiab] OR sexual‐harassment [tiab] OR domestic‐violence [tiab] OR child‐abuse [tiab] OR IPV [tiab] OR physical‐violence [tiab] OR sexual*‐abuse [tiab] OR traffick* [tiab] OR transphobia [tiab] OR homophobia [tiab] OR women [tiab] OR child* [tiab] OR gender [tiab] AND econ* [tiab] OR poverty [tiab] OR empower* [tiab] OR norm* [tiab] OR social [tiab] OR relation* [tiab]

### 4.5 Analysis and presentation

#### 4.5.1 Report structure

The report follows Campbell's guidelines for structure for EGM: an abstract, plain language summary, background, methods, results, and authors' conclusions.

#### 4.5.2 Filters for presentation

We included the following five filters for presentation for our EGM:

- Region where study was conducted
- Type of ICT device used by the intervention
- Specific target population
- Sponsor organization
- User of the intervention ICT device (primary vs intermediary target)

See Supporting Information: Appendices for Coding Sheet Examples (for purposes of analyis, but are not all included as final filters for presentation in the EGM).

##### Dependency

Define the unit of analysis for primary studies. Define whether each item represents a report or a study, and what will be done when there are multiple reports for a single study, or when a report covered multiple studies.

We included individual studies with multiple reports as a single study in our aggregate count of eligible studies, although we provided citations for separately published articles pertaining to the same study (e.g., published protocols). This includes any studies that are included in more than one systematic review

### 4.6 Data collection and analysis

#### 4.6.1 Screening and study selection

EPPI ‐Reviewer was used to screen and code studies and articles. Independent reviewers first independently screened studies' titles and abstracts. Disagreements between reviewers were resolved by consensus. Potentially eligible studies were then retrieved in full text and these full texts were reviewed for eligibility, again using two independent reviewers. Disagreements between reviewers were resolved via discussion and consensus. The completed review includes a table of studies excluded at the full text level of screening and provide rationale for each exclusion decision. We also include a PRISMA flow chart to report the screening process and results (PRISMA‐P, Group et al., 2015).

#### 4.6.2 Data extraction and management

Two review authors, also unblinded to author or journal information, independently extracted information from the included trials. This information was recorded in a data‐extraction form that was piloted before initiation of the review. Discrepancies between reviewers regarding data extraction were resolved by consensus or if required via a third reviewer (which did not occur).

In addition to the standard categories used in coding of studies such as year of study, setting and other contextual features, target population(s), study method(s), sample sizes outcomes, etc., we included categories regarding classification of ICT (e.g., mobile phone, tablet, web‐based computer, etc.), general characteristics of, including age categories of ICT users, connectivity and other ICT enabling conditions (e.g., literacy), type of intervention: (1) Prevention: (primary, secondary, tertiary) or (2) Response (technical sector: e.g., health, justice, psycho‐social, education, etc.). We drew from commonly and generally accepted frameworks for analyzing SGBV for children (INSPIRE), and for women (e.g., RESPECT; Essential Services Package for Women and Girls Subject to Violence); Minimum Standards for Prevention and Response to Gender‐based Violence in Emergencies.

See Supporting Information: Appendices for a copy of the coding sheet with illustrative categories.

#### 4.6.3 Tools for assessing risk of bias/study quality of included reviews

The Risk of Bias Table is included below as Table 1.

**Table 1:** Risk of Bias Analysis Table

Citation	ROB tool	Score	Areas of potential bias	Comments
Anderson et al. (2019)	AMSTAR‐2	7/9 Low Risk	Controls not explicitly defined, excluded studies not listed	Study controls were mentioned in text, but not fully defined. Exclusion reasons stated, but not specific excluded studies.
Eisenhut et al. (2020)	AMSTAR‐2	3/9 High Risk	No discussion of controls or outcomes, nor study selection criteria, insufficient information regarding about data extraction. Did not include list of excluded studies or information related to ROB or study selection/extraction in duplicate.	Review methods established prior to before start of the review, explicit search strategy and detailed information on included materials, but all other items lacking: no information related to study design selection, risk of bias, or duplicate tasks for screening/data extraction.
Hayes (2014)	AMSTAR‐2	1/9 Not Systematic	Included studies described in sufficient detail (Item 8), all other items missing from report.	The author explicitly states that this report is a desk review and not a systematic review in the methods section of the report.
Linde et al. (2020)	AMSTAR‐2	7/9 Low Risk	Controls not explicitly defined, excluded studies not listed	Did not include an explicit discussion of controls of studies, nor list studies that were excluded (exclusion reasons indicated).
Brody et al. (2021)	Cochrane ROB Tool for RCTs	Low Risk	n/a	n/a
Clark et al. (2020)	Cochrane ROB Tool for RCTs	Some concerns	Measurement and analysis of outcomes	Measurment of outcomes insufficiently described in report.
Decker et al. (2020b)	Cochrane ROB Tool for RCTs	Low Risk	n/a	n/a
Ampt et al. (2020b)	JBI Manual of Evidence Synthesis	9/10 Low Risk	No statement locating researchers culturally or ethically	n/a
Bartels et al. (2019)	JBI Manual of Evidence Synthesis	9/10 Low Risk	No statement locating researchers culturally or ethically	n/a
Mishori et al. (2017)	JBI Manual of Evidence Synthesis	9/10 Low Risk	No statement locating researchers culturally or ethically	n/a

##### Quality of evidence assessment of reviews

The AMSTAR 2 tool (Shea et al., 2017) was used to assess the quality of reviews and assess risk of bias. Quality of Evidence/Risk of bias assessment was conducted by two researchers in duplicate with any discrepancies resolved by discussion.

##### Quality of evidence assessment of studies

Quality of evidence for RCT's was assessed using Cochrane's Risk of Bias Tool. Qualitative data was assessed using the JBI Critical Appraisal Took for Qualitative Evidence Aromataris & Munn, 2020. Two researchers conducted a quality of evidence assessment to resolve through discussion any discrepancies.

#### 4.6.4 Methods for mapping

We used EPPI‐Reviewer and EPPI‐Mapper to generate our final EGM.

## 5 RESULTS

### 5.1 Description of studies

#### 5.1.1 Results of the search

The attached PRISMA flow chart graphically depicts the numbers of references identified, and then excluded through screenings, as well as the final number of eligible studies (10) identified for coding.
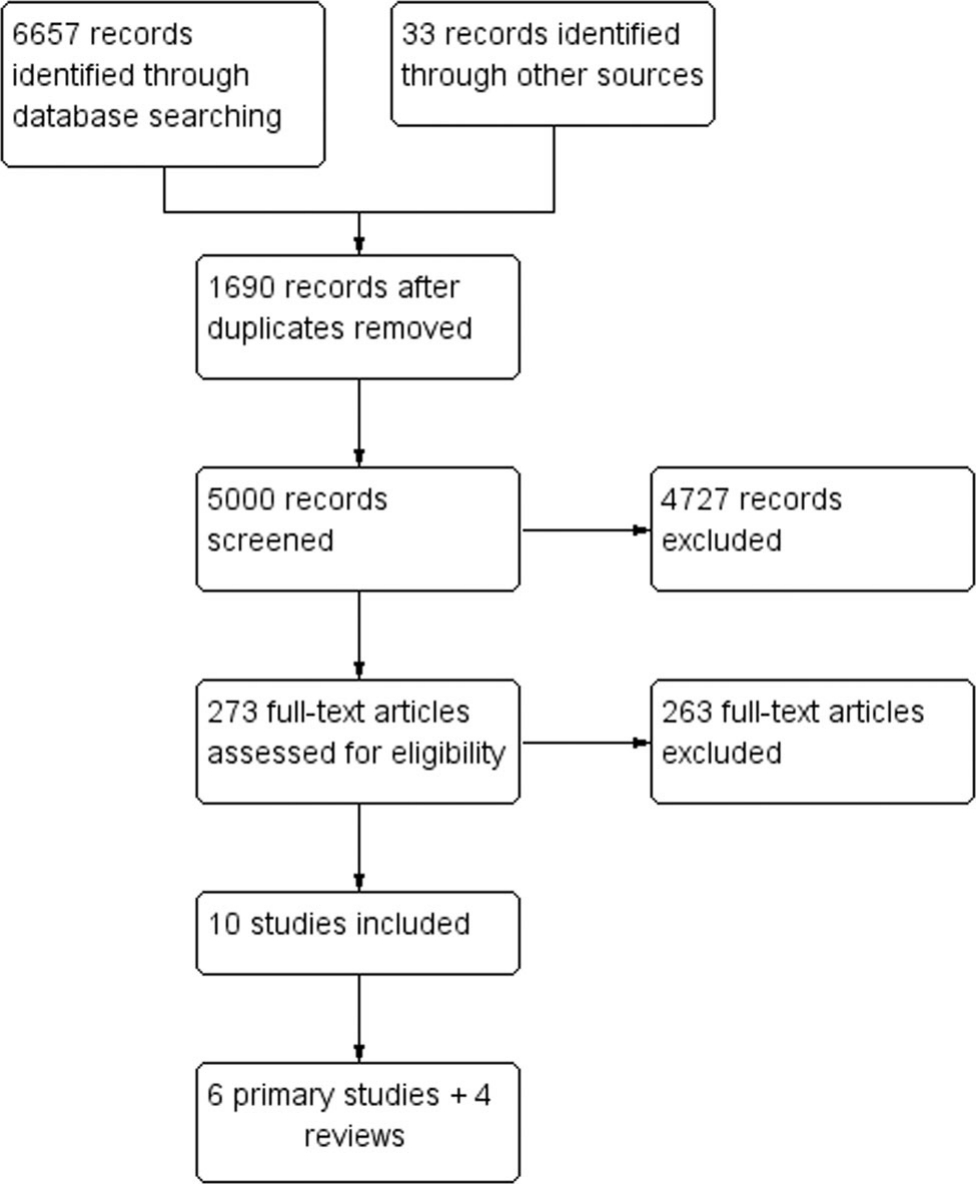


The screening process resulted in 10 records qualifying as eligible for coding. (16 citations are in the “Included” studies, but these may included multiple publications regarding the same study or review such as a protocol.) Of the 10 eligible studies, 4 were reviews, 3 of which were characterized in their titles as “systematic reviews,” but 1 of these 3 was actually a scoping review of mobile phone applications rather than peer‐reviewed publications. The fourth was a literature or scoping review presenting case studies. The remaining six were individual studies, of which three could be truly understood as presenting ICT‐ linked SGBV “impact” results. Four of the six studies included protocols that had been published separately from the results of the study (and may have been modified in the report of the final results of the study), and therefore are not counted separately (Ampt et al., 2020b; Brody et al., 2021; Clark et al., 2020; Decker, Wood,  Hameeduddin, et al., 2020). One of the protocols separately published included a detailed description of the process for adapting an intervention deemed effective in HIC to an LMIC context (Decker, Wood, Kennedy, et al., 2020). One of the *reviews* published a protocol separately (Anderson, Krause, et al., 2019). The search extended into Portuguese and Spanish language databases (languages spoken by the authors).d/or applications conducted in HIC, but all four include at least one study/application conducted in an LMIC, in accordance with the eligibility criteria for inclusion. See Summary of findings Table 2.

Figure 2 provides a summary of the search and screening process to identify eligible studies for inclusion. The electronic search of relevant academic databases returned 6657 records. An additional 33 records were added from citations in the systematic reviews that did not turn up in the database searches. 1690 duplicates were excluded. Two rounds of title and abstract screenings returned 273 records for screening of full texts. Of these, 10 studies met the inclusion criteria for the EGM. All included studies were in the English language. Also, from the 273 screened for full‐text analysis, we identified an additional 31 studies from HIC that would have otherwise been included had they come from LMIC contexts. We identified these 31 HIC studies to provide context, comparisons, and relevant insights for LMIC in the Discussion section below.

We include a list of the key excluded studies that the reader might reasonably expect to find included in an EGM examining the use of ICT to prevent an/or respond to SGBV against women and children. The main reasons for exclusions included: (1) the study or studies occurred only in an HIC context; (2) intermediate outcomes of interest contained in the study (informed by the evidence‐based RESPECT and INSPIRE frameworks) were not explicitly linked with SGBV or GBV; (3) the technology used was not “ICT” as defined under the protocol; (4) the study addressed “technology‐facilitated SGBV/GBV,” a category explicitly excluded in the search strategy (e.g., online stalking;) and (5) the article did not report on the results of a study (but was rather an editorial or commentary). Due to the large number of studies screened at full text, we do not provide a comprehensive list of all studies excluded after the full text screening, but this list is available upon request.

### 5.2 Synthesis of included studies

Unless otherwise specified, we refer to the primary study with the published results.

See Summary of findings Table 2 (primary studies)

See Summary of findings Table 3 (“systematic” reviews)

### 5.3 Resulting evidence from studies

There is not yet a single body of evidence of methodologically rigorous studies from which generalizations on impact can be made. There is also not yet a cluster of evidence demonstrating the *consistent* impact of a particular type ICT intervention for preventing or responding to SGBV against women and children in LMIC.

With that said, several studies, based upon the rigor of methodology and outcomes, demonstrated promising results as a baseline warranting further research, particularly in different contexts. One study that demonstrated impact in preventing (and responding) to SGBV was the Decker, Wood, Hameeduddin, et al. (2020) study evaluating how an ICT safety decision aid app (MyPlan), proven effective in HIC contexts, can be adapted to be effective in an LMIC context, specifically Kenya. Intervention groups reported increased usefulness of safety strategies, increased in resilience and significant decreases in the risk for lethal violence compared to the control group. Intervention participants also demonstrated immediate post‐intervention safety preparedness relative to control participants. Both intervention and control groups in the Kenya study reported reductions in IPV (Decker, Wood, Hameeduddin, et al., 2020). Current efforts are underway to study the impact of using MyPlan in other LMIC contexts including Somalia. (Glass, 2017)

The Clark et al. (2020) study, an RCT, highlighted how ICT (SMS and IVR), although not studied in isolation with a control, can effectively contribute to a multi‐media approach toward reducing SGBV (IPV) against Nepalese women. The Bartels et al. (2019) study demonstrated how ICT (using the SenseMaker M&E tool) could be used to facilitate gathering the impressions of women regarding the quality of, and important criteria for, SGBV/GBV services in Lebanon (humanitarian setting). These impressions can be used to inform the quality of SGBV services (and improve access) although route of exploration was not an objective of the Bartels study. The Mishori et al. (2017) study highlighted how ICT (using MediCapt) could be used as a tool by clinicians and prosecutors for collecting forensic information in cases of SGBV. Whether the forensic evidence collected contributes to medical, legal, and human rights outcomes is under current study (Mishori et al., 2017, p. 150).

The four reviews did not present consistent conclusions regarding impact. Two of the four reviews looked at existing eHealth and/or mHealth interventions/tools and their impact on IPV. The Linde et al. (2020) review, evaluating the effect of “eHealth interventions compared with standard care on reducing IPV, depression, and post‐traumatic stress disorder (PTSD) among women exposed to IPV,” as well as secondary outcomes of “type‐specific IPV,” concluded that there “is not evidence from randomized trials of a beneficial effect of eHealth interventions on IPV.” The Linde et al. (2020) review recommended “more high‐quality trials” and “harmonizing outcome reporting in IPV trials by establishing core outcome set.” Similarly, Anderson, looking at mHealth interventions, concluded that evidence for efficacy of mHealth interventions compared to conventional IPV was “limited” (Anderson, Krause, et al., 2019). The Eisenhut et al. (2020); and Hayes (2014) reviews were more of scoping or landscaping reviews which did not include reviewing methodologically rigorous studies of impact.

#### 5.3.1 Types of ICT intervention

The kinds of ICT‐facilitated SGBV prevention and/or response interventions included:

- SMS and IVR messaging (Ampt et al., 2020b; Brody et al., 2021)
- Digital safety decision algorithm tool Decker, Wood, Hameeduddin, et al., 2020)
- Recording experiences of women (monitoring and evaluation) (Bartels et al., 2019)
- Radio drama with listener engagement through IVR and SMS (Clark et al., 2020)
- Forensic and clinical documentation of violence (Mishori et al., 2017)

Types of interventions reported in the four (4) reviews (including those taking place in HIC contexts):

- web‐based tutorials
- computer‐based assessment/screening
- tablet‐based education modules
- email delivered links to online resources
- communication network to connect women with information regarding SGBV
- toll‐free counseling and support
- hotline support
- interactive map showing incidents of SGBV
- using SMS, email, and social media to report sexual harassment (evidence collection)
- problem‐solving training
- gamification (for children and adolescents) to change attitudes around gender and SGBV/GBV)
- mental health support; web‐based CBT.

Modes of ICT included:

- radio
- cell phones
- smart phones
- tablets
- computer/web

#### 5.3.2 Indicators/outcomes measured

Indicators for outcomes measured fell into two categories: (1) impact indicators, which also include intermediate outcomes for SGBV prevention as outlined by the evidence‐based RESPECT and INSPIRE frameworks; and/or (2) formative indicators, which include process outcomes related to usability and feasibility of the ICT tool. Such formative outcomes ideally reflect the Principles of Digital Development. As discussed below, the process outcomes are an essential ingredient, without which achieving intended SGBV‐related impact may be undermined.

Of the six primary studies, four included in their original methodologies outcome measurements, either as primary or secondary outcomes, explicitly related to SGBV/GBV prevention or response (Ampt et al., 2020b; Brody et al., 2021; Clark et al., 2020; Decker, Wood, Hameeduddin, et al., 2020). Two of the systematic reviews included studies with explicit SGBV/GBV outcomes (Anderson, Krause, et al., 2019; Linde et al., 2020). While the Ampt et al. (2017), and Ampt et al. (2020b) articles included GBV explicitly as one of its intervention domains, the final study results made no explicit mention of SGBV/GBV (Ampt et al., 2020b).

Four of the six primary studies identified measured outcomes that can be characterized as an intermediate outcome for preventing SGBV (Ampt et al., 2020b; Brody et al., 2021; Clark et al., 2020; Decker, Wood, Kennedy, et al., 2020, 2020; Decker, Wood, Hameeduddin, et al., 2020). Ampt indicated the intermediate outcomes in the original published methodologies (Ampt et al., 2017, 2020b), but not in the publication of the final results (Ampt et al., 2020b). Such intermediate outcomes include relationship control and skills strengthened (including conflict resolution); environments made safe (e.g., reduced alcohol use); safety preparedness; and transformed attitudes, beliefs, and norms (e.g., acceptability of GBV [World Health Organization, 2019a]).

The INSPIRE framework of the seven strategies for preventing violence against children is not mentioned in this section as none of the eligible studies explicitly related to children and child sexual abuse (World Health Organization, 2016).

Of the systematic reviews, Anderson, Krause, et al. (2019); and Linde et al. (2020) included studies with explicit SGBV‐specified outcomes or RESPECT framework intermediate outcomes. The goal of the Linde systematic review was to “estimate the effect of eHealth interventions compared with standard care on reducing overall IPV (physical, sexual, or psychological violence), type‐specific IPV, depression, and PTSD among women exposed to IPV” (Linde et al., 2020). The only LMIC studies included in the Linde et al. (2020) review were from Nepal (Clark et al., 2020) and Kenya (Decker, Wood, Hameeduddin, et al., 2020), both are which are synthesized separately under this EGM (although the final citation of results for Kenya study was not included in the Linde review, and we had to contact the author for the final publication of results).

Linde summarized the impact of all the included studies (which included studies conducted in HIC) as finding “no evidence from randomized trials of a beneficial effect of eHealth interventions on overall IPV; physical, sexual, or psychological violence; or depression and PTSD. However, the types of outcomes and how they were measured were very heterogenous across trials, which limited the possibility of pooling results and identifying patterns across studies” (Linde et al., 2020). The Anderson systematic review included only one study connected in an LMIC, specifically a protocol from Nepal (Brody et al., 2018), the final study results of which were obtained from the author (Brody et al., 2021). In addition to other outcomes, the Linde et al. (2020) review screened for studies with the reduction in IPV experiences or effects as outcomes.

Four of the primary studies reported on SGBV response outcomes (Bartels et al., 2019; Brody et al., 2021; Decker, Wood, Hameeduddin, et al., 2020; Mishori et al., 2017). These indicators included: perceptions and feeling connected with accessing SGBV services and their perceived benefits (Bartels, 2019); utilization of referral services (Brody et al., 2018); responses to, and acceptance of SGBV/IPV;

Two of the primary studies used formative process indicators regarding the development of the ICT component of the intervention focusing on ensuring participation of the users in the design of the intervention: usability/user experience, acceptability, and feasibility (Decker, Wood, Kennedy, et al., 2020; Mishori et al., 2017).

Although extremely informative, two of the reviews were primarily descriptive in nature, serving more as landscape reviews of different kinds of uses of ICT for SGBV. Neither review reported the results of methodologically rigorous studies of impact (Eisenhut et al., 2020; Hayes, 2014).

#### 5.3.3 Target population (beneficiaries and users)

The target populations, or the populations to ultimately benefit from the ICT interventions in all six individual studies were women (and girls). Two of those studies specifically targeted female entertainment workers. One of the individual studies targeted refugee women and girls (as beneficiaries of SGBV/GBV services). None of the included studies examined SGBV against children, boys, or LGBTQ.

Users of the ICT included women themselves (*n* = 3); community (members/women and men) (*n* = 1); service provider/M&E and women/girls (*n* = 1) and service providers only (*n* = 1).

#### 5.3.4 SGBV context/setting

All eligible included studies took place in an LMIC context. Countries represented in the primary studies include Cambodia (Brody et al., 2021; Kenya (Ampt et al., 2020b; Decker, Wood, Hameeduddin, et al., 2020), Lebanon (Bartels et al., 2019; Nepal (Clark et al., 2020) and the Democratic Republic of Congo (DRC) (Mishori et al., 2017).

LMIC countries and regions represented within the reviews include:

- Afghanistan, Cambodia, Costa Rica, DRC, Egypt, El Salvador, India, Nepal, Nicaragua, Sierra Leone, and Palestine (Hayes, 2014).
- Sub‐Saharan Africa, Middle East and Northern Africa (MENA), South Asia, Latin America and the Caribbean (LAC), and Central Asia (Eisenhut et al., 2020).
- Cambodia (Anderson, Krause, et al., 2019).
- Kenya and Nepal (Linde et al., 2020).

Of the studies included, two of the primary studies and two of the reviews focused on IPV contexts (Anderson, Krause, et al., 2019; Clark et al., 2020; Decker, Wood, Kennedy, et al., 2020; Linde et al., 2020; (b). Two primary studies focused on the female entertainment industry, that is, female sex workers (Ampt et al., 2020b; Brody et al., 2021). Two primary studies focused on humanitarian/emergency contexts (Bartels et al., 2019; Mishori et al., 2017). The literature review of case studies addressed multiple contexts (Hayes, 2014).

None of the eligible studies focused on children or any sort of CSA or LGBTQ populations.

Our searches also revealed a small number of papers that discussed the potential role ICT‐based interventions may play in preventing and responding to SGBV in LMIC in the context of COVID‐19 pandemic lock‐downs and displacement/forced migration settings. These papers were editorials or theoretical discussions that did not refer to any specific evidence or intervention. Despite this, they demonstrate a growing interest for using ICT in LMIC by SGBV experts and researchers. Though none of these papers met the inclusion criteria, they contemplate how policymakers and experts may leverage ICT‐based interventions in specific contexts, such as refugee camps, to prevent and respond to SGBV. More systematic and methodologically rigorous research to understand the diversity of ICT ecosystems in LMIC could better contextualize these discussions.

Link (URL) to the online interactive EGM: https://onlinelibrary.wiley.com/pb-assets/assets/18911803/PhilbricketAliaFINALEGM-1662372530.html

#### 5.3.5 Study design

Four of the six primary studies reported on randomized control trials (RCTs) (Ampt et al., 2020b; Brody et al., 2021; Clark et al., 2020; Decker, Wood, Hameeduddin, 2020). One of the systematic reviews included studies that were all RCTs (Linde et al., 2020). One review included RCTs, but also included non‐RCTs (Anderson, Krause, et al., 2019). One review, although characterized as a “systematic review” appeared to be more of a scoping review, reporting on the landscape of available apps, and not on studies evaluating the effectiveness of those apps (Eisenhut et al., 2020). The fourth review included case studies collected through a literature review, including references to evaluations conducted by organizations connected to those apps, thus presenting potential conflicts of interest regarding the presentation of evaluation results (Hayes, 2014).

One of the included studies could be characterized as solely qualitative (Hayes, 2014). However, the Ampt study published separate results of a qualitative portion of the study (Ampt et al., 2020b). Four of the included studies are characterized as, or included studies with mixed methods designs, including the final Ampt publication of the results of the WHISPER intervention (Ampt et al., 2020b; Bartels et al., 2019; Brody et al., 2021; Eisenhut et al., 2020). Two of the reviews included references to multiple types of studies (Anderson, Krause, et al., 2019; Linde et al., 2020.)

#### 5.3.6 Primary prevention, response or both

All interventions reported in the primary studies could be characterized as an ICT‐facilitated response to SGBV. As discussed under the protocol to this EGM, there are three types of prevention: primary, secondary, and tertiary. Both secondary and tertiary prevention interventions can sometimes be characterized as response interventions because they occur after an incident of SGBV has occurred. Secondary prevention is sometimes focused on preventing specific violence from continuing or escalating. Tertiary prevention focuses on minimizing the impact of violence and incidence of future violence.

Those interventions, however, focusing on *primary prevention*, preventing SGBV before it even occurs, are significantly less represented. None of the eligible studies focused exclusively on primary prevention. Most (6) studies focused both on elements of prevention and response (Ampt et al., 2020b; Anderson., Krause, et al., 2019; Brody et al., 2021; Clark et al., 2020; Decker, Wood, Hameeduddin, et al., 2020; Eisenhut et al., 2020; (b). Two studies focused on SGBV response only (Bartels et al., 2019; Mishori et al., 2017). While Eisenhut et al. (2020) mentions applications for SGBV “avoidance,” most of the applications presented are primarily for response. Mishori et al. (2017) discusses using ICT for collecting evidence of SGBV.

### 5.4 Risk of bias in included reviews and quality of evidence for individual studies

The four reviews included were analyzed for risk of bias and quality of evidence using AMSTAR‐2 tool for systematic reviews (Shea et al., 2017). Two reviews (Anderson, Krause, et al., 2019; Linde et al., 2020) scored high on the AMSTAR‐2 tool presenting low risk of bias. The third review (Eisenhut et al., 2020) demonstrated some risk of bias, as the review did not include information related to study design selection criteria, nor information as to whether or not analysis and extraction were performed in duplicate. However, this review did not search for published peer‐reviewed studies in scientific databases, but searched for mobile phone applications specific to SGBV directly though mobile application hosting platforms (i.e., *app stores*). The forth review (Hayes, 2014) was not truly systematic in nature and, as a result, scored low on the AMSTAR‐2 tool. However, (Hayes, 2014) is better characterized as a desk review of several case studies. The authors were cognizant of this distinction from a traditional systematic review in their methodology, which was otherwise sound. The review therefore met the inclusion criteria for this EGM.

Though optional, the six individual studies were analyzed for quality of evidence using several tools. The three included RCTs where analyzed using the Cochrane Tool for Risk of Bias for RCTs. Two RCTs (Brody et al., 2021; Decker, Wood, Hameeduddin, et al. (2020) were at low risk of bias according to the Cochrane analysis, while Clark et al. (2020) presented some concern of bias regarding the measurement of its outcomes. The remaining three studies were all qualitative (feasibility studies) and analyzed using the Critical Appraisal Checklist for Qualitative Research from the JBI Manual of Evidence Synthesis (Aromataris & Munn, 2020). All three studies scored highly for quality of evidence with low risk of bias.

### 5.5 Additional dimensions (if applicable)

#### 5.5.1 Theoretical basis

Deemed integral to the foundation and quality of a study in the opinion of many researchers is whether an intervention is based upon sound validated theory, such as a behavior change theory (Philbrick, 2013, p. 27). Six of the total ten studies made reference to some sort of theoretical underpinning for the intervention. (Ampt et al., 2020b; Brody et al., 2021; Clark et al., 2020; Decker, Wood, Kennedy, et al., 2020; Eisenhut et al., 2020; Mishori et al., 2017).

#### 5.5.2 Theory of Change

Articulating a clear and cogent Theory of Change or logic model is also a characteristic of what many academics consider quality interventions. Four of the six included primary studies included clear Theories of Change in either the protocol (if separately published) or final report of the results (Ampt et al., 2020b; Brody et al., 2021; Clark et al., 2020; Mishori et al., 2017).

#### 5.5.3 Unintended consequences addressed

Peer review of the protocol for this EGM emphasized the important of disclosing and discussion unintended consequences connected with using ICT. The most common, and perhaps most dangerous consequence when using ICT for a highly sensitive subject like SGBV is inadvertent disclosure of personal information and breach of confidentiality. While unintended consequences should not be a disqualifying factor preventing the use of ICT to facilitate interventions, they must be recognized and factored into the intervention design.

Four of the ten included studies addressed unintended consequences connected with using ICT in connection with SGBV (Ampt et al., 2020b; Eisenhut et al., 2020; Hayes, 2014; Mishori et al., 2017). Participants in the Ampt study expressed some concerns over potential breaches of privacy and personal safety from using the ICT‐based intervention. While the authors described what these concerns were, they did not specify solutions to these concerns (Ampt et al., 2020b). The review by Eisenhut *et alia* and the Hayes paper both discussed issues with mobile device sharing in LMIC (i.e., households often share a single device) and how intervention developers cannot assume that women will have exclusive access to their mobile devices, and that this could conceivably elevate risk of IPV in some scenarios (Eisenhut et al., 2020; Hayes, 2014). Mishori et al., which detailed the formative research for an ICT‐based forensic data collection tool in conflict settings, discussed the need for strict data security to protect survivor privacy and ensure quality of evidence. While the authors discussed solutions to these risks as on‐going, they pointed out that current paper‐based forensic evidence is arguably *more* vulnerable in conflict settings as is, suggesting that a risk‐benefit give and take is often necessary when considering current contexts and the potential role of ICT‐based alternatives. (Mishori et al., 2017).

#### 5.5.4 Sustainability

Of the included studies (both individual and reviews)**,** four addressed issues of sustainability connected with ICT‐facilitated interventions (Bartels et al., 2019; Brody et al., 2021; Hayes, 2014; Mishori et al., 2017). Bartels et al. discussed how lower levels of literacy and familiarity with technologies such as tablets and mobile phones greatly affected the ease with which end‐users could engage with their ICT‐based intervention, and that this was a significant potential barrier to scale up (Bartels et al., 2019). The authors of the Mishori study discussed that sustainability was a key factor in their intervention design, especially how this relates to future scale up and ensuring that the software created for their intervention would eventually transition to open‐source (i.e., be freely available for implementors to use without issues of copyright, for example) (Mishori et al., 2017. The Hayes paper, which described several case studies from LMICs, indicated that access to sufficient funding and resources was often the greatest barrier for ICT‐based interventions longevity and sustainability (Hayes, 2014).

#### 5.5.5 LMIC‐based authors/researchers

We also believed it was important to identify the included published studies which had at least one author/researcher based in an LMIC. Five (5) of the ten included studies included at least one LMIC‐based author/researcher (Ampt et al., 2020b; Bartels et al., 2019; Brody et al., 20211; Decker, Wood, Hameeduddin, et al., 2020; Mishori et al., 2017).

## 6 DISCUSSION

### 6.1 Summary of main results

Broadly, there is an, overall dearth of methodologically rigorous studies conducted in LMIC examining the use of ICT for the prevention of, and response to SGBV. While there were several studies addressing SGBV against women, we did not identify any eligible studies specifically addressing SGBV against children (i.e., child sexual assault or “CSA”) nor against LGBTQ populations (i.e., targeted attacks against transgender or gender non‐conforming persons). There does appear, however, to be an upward trend in LMIC to study how ICT may be best leveraged to address SGBV against women and to a lesser extent girls. There is also a limited number of ongoing studies in LMIC that use ICT for addressing VAC/CSA, including gamification for adolescents to change underlying attitudes and beliefs and digital parenting interventions. Overall, we found a scarcity of studies measuring primary prevention, with most uses of ICT focused on secondary and tertiary prevention of SGBV (otherwise characterized as response interventions).

While there is no single body of evidence demonstrating the *consistent* impact of a particular type of ICT intervention for preventing or responding to SGBV against women and children in LMIC, we noted several promising completed individual studies.

One study that demonstrated impact in preventing and responding to SGBV was the Decker, Wood, Hameeduddin, et al. (2020) study evaluating how an ICT safety decision aid app (MyPlan), proven effective in HIC contexts can be adapted to be effective in an LMIC context, specifically Kenya. Intervention groups reported increased use of safety strategies, increases in resilience and significant decreases in the risk for lethal violence when compared to the control group. Intervention participants also demonstrated immediate post‐intervention safety preparedness relative to the control. Both intervention and control groups in the Kenya study reported reductions in IPV (Decker, Wood, Hameeduddin, 2020, p. 1).

While safety apps like MyPlan can be strategic in reducing SGBV by helping potential SGBV targets (of both sexes) in evaluating their circumstances and avoid situations in which they have a heightened risk to SGBV, they do not necessarily address the underlying attitudes and norms fueling SGBV. The most promising type of intervention involving ICT for primary prevention would be an intervention that has an objective related to changing gender and social norms (toward SGBV). This would include changing underlying attitudes toward SGBV acceptability by both the perpetrator and the victim/survivor. In some cultures, women and girls (and others) may think that SGBV is something to which they must acquiesce and resign themselves. Interventions that target changing gender and social norms go beyond increasing knowledge and targetthe root causes of most SGBV. Accordingly, interventions targeting young people (adolescents) and developing social norms that reject SGBV, serve as potentially the more promising interventions for primary prevention of SGBV. Ongoing studies around gamification (BREAKAWAY Guatemala Pilot Study) and digital parenting through the Parenting for Lifelong Health program (Digital Parenting Studies) could confirm the strategic use of ICT for contributing to primary prevention of SGBV.

The Clark et al. (2020) study seems to infer that changes to underlying attitudes regarding SGBV require a multi‐media approach involving both community discussions, reinforced by ICT messaging.

Formative studies that measure usability, feasibility and elicit participatory input from an ICT intervention's targeted user population are also necessary ingredients for effective ICT design and implementation They are as equally as important as studying and measuring impact because they influence the uptake and sustainability of an intervention, a consideration we did not find often addressed nor emphasized in those studies and reviews we deemed eligible for this EGM. Formative studies are acutely pertinent because local contexts and enabling environments for ICT are varied and cannot be generalized, particularly in LMIC.

One such study (Mishori et al., 2017) described the formative process, including needs and resource assessments, end‐user feasibility assessments and field pilot testing, for an ICT‐based sexual violence data collection tool (MediCapt) in the DRC. While most studies that turned up in our search described the use of ICT in prevention of, and response to SGBV originated in HIC (albeit not eligible), we identified only one study demonstrating a formative process for how ICT interventions proven effective may be adapted form a HIC to an LMIC context (Decker, Wood, Kennedy, et al., 2020).

While we excluded studies and interventions conducted in HIC from eligibility, we did find that the reviews containing a disproportionate number of studies conducted in HIC included interventions in the areas of addressing intimate partner violence (IPV), and evaluating the effectiveness, acceptability, and “suitability of ICT” for addressing different aspects of awareness, screening, prevention, treatment, and mental health. A number of these studies explored how current mHealth web‐based eHealth interventions could be leveraged to address SGBV. While these HIC‐based studies published minimum evidence of effectiveness (and in the case of Linde et al. (2020) no evidence of effectiveness in using mHealth/eHealth) in addressing SGBV (with the exception of safety apps), they do provide a foundation and some limited promising results to further explore, particularly for adaptation to LMIC contexts (see, e.g., Anderson, Krause, et al., 2019; Braithwaite & Fincham, 2014; El Morr 2020, Glass, 2017; Glass et al., 2021; Rempel, 2019).

Measuring effectiveness in term of absolute changes in levels of SGBV (e.g., IPV incidence) with validated tools, can be difficult and requires longitudinal resources. Effectiveness studies, however, can also measure evidence‐based intermediary outcomes such as those outlined under the RESPECT and INSPIRE frameworks published by the World Health Organization. Validated tools for measuring different impact outcome variables, whether primary (changes in SGBV prevalence), intermediary (e.g., changes in women's empowerment, or changes in reported cases of sexual violence) are varied. Clear consensus for standardized indicators and measurement tools does not yet exist. The research we observed during our review reflected this, and at least one of the reviews explicitly noted the lack of harmonized outcome indicators (Linde et al., 2020). Ironically, the observation about the lack of a lack of homogeneity among the outcome measurements, an obstacle to making analytical comparisons, was also noted in a review of ICT used for SGBV in HIC as well (El Morr, 2020, p. 1)

Of the studies that measured some sort of intermediary “impact,” there appears to be more studies measuring how ICT may increase women's “empowerment,” strengthen relationships and contribute to conflict resolution. There also seems to be a small cluster of studies gauging ICT end‐user acceptance and feasibility, both necessary for adoption and use of ICT facilitated interventions contributing to SGBV prevention and/or response outcomes.

See Figure 2 RESPECT Preventing Violence Against Women Framework (World Health Organization, 2019a).

See Figure 3 INSPIRE Seven Strategies for or Preventing Violence Against Children (World Health Organization, 2016).

### 6.2 Areas of “evidence clusters”

As mentioned, even though there was a low number of included studies, certain areas emerged based on a few individual studies which appear promising for future research efforts in LMIC. These areas, however, cannot be characterized as “clusters of evidence.” All of the eligible studies focused on prevention and/or response to SGBV against women and girls, although several limited the target group to women above a certain age. All of these studies were diverse in outcomes, measurements, and design.

It is important to note that the bulk of the evidence from the systematic reviews (that included both HIC and LMIC‐based interventions was from HIC settings and reported that the heterogeneity of study design and IPV outcomes made generalization and meta‐analysis difficult across studies. Results from Clark et al. (2020) did not report significant changes in IPV between the control and intervention, though the intervention was associated with some decreases of certain forms of IPV at endline. (Clark et al., 2020.) The Clark study, however, did not isolate the digital ICT component for evaluation, but rather evaluated a combined multi‐media intervention.

The types of ICT intervention represent a broad spectrum (SMS, IVR, web‐based decision‐algorithms, etc.) and no one single type of intervention emerges as predominant in the body of eligible studies from LMIC. However, studies from HIC, although not included as eligible for this EGM, seem to suggest that the use of SMS to be multifaceted and leveraged for different purposes related to both prevention and response: underscoring communications for changing gender norms, building capacity of service personnel, connecting survivors to each other and with services, reporting incidents, mental health support, etc. Adapting those interventions proven effective in HIC for LMIC contexts, could be strategic. The MyPlan adaptation protocol could serve as a model guide, as well as SVRI's Adaptation Guide under development (Sexual Violence Research Initiative, 2021).

Targets, in terms of users and beneficiaries, primarily included women. However, including potential perpetrators of SGBV as targets (in terms of users) may be strategic in realizing prevention goals. The Clark et al., 2020 study is an example which included communities and couples. The ongoing Breakaway study, targeting both sexes of adolescents (boys and girls) may be informative for future targeted interventions.

### 6.3 Areas of major gaps in the evidence

Our review reveals that there are few methodologically rigorous studies of ICT‐facilitated interventions for the prevention and/or response of SGBV in LMIC, especially relative to the number of studies conducted in HIC. There is a dearth of studies utilizing RCT methodologies, the “gold standard” for demonstrating evidence of effectiveness. While we did identify some evidence looking at how ICT can be facilitated for interventions to prevent and/or respond to SGBV in LMIC, there were overall very few studies. There is not yet a strong consistent body of evidence connected with using ICT for SGBV in LMIC.

The reviews of ICT interventions indicated that there is not yet a consensus nor harmonized set of outcome indicators relating to using ICT in SGBV prevention and/or response. We observed some studies measuring intermediate outcomes as defined under the RESPECT framework, but a more explicit linking of these intermediate outcomes to SGBV primary prevention would strengthen the evidence base for using ICT for SGBV prevention and/or response. We also noted studies that measured changes in attitude regarding IPV, but not with a consistency that would allow for characterizing them as “clusters.” Further, there is a gap in the number of studies that isolate and compare the ICT component in the intervention target group with a control target group in which ICT is not used, such as in the Clark et al. (2020) study.

As evidenced by the lack of included eligible studies, research is needed to examine the role that ICT can play in addressing SGBV against children, adolescents, and LGBTQ populations in LMIC. This gap is highlighted by the increasing proliferation of smart phone use amongst children and youth in LMIC. One such explanation for the dearth of studies examining the use of ICT regarding SGBV against children may be the immense challenges securing an Insitutional Review Board (IRB) approval for any study involving children as subjects. There is also an exponential growth of technology‐facilitated SGBV that is not fully understood in LMIC, especially as this relates to human trafficking (see, e.g., Segrave & Vitas, 2017; Van Reisen et al., 2017). As technology‐facilitated SGBV violence has become a common trait connected with trafficking, more efforts to study how ICT can be used to counter and prevent it are warranted. Interventions with the objective of addressing technology facilitated violence were excluded from this EGM.

In the same vein, while there are a number of interventions targeting SGBV response (secondary and tertiary prevention), noticeable gaps exist related to how ICT can contribute to addressing the underlying reasons for SGBV for primary prevention (e.g., addressing gender and social norms.).

We identified two studies that examined how ICT can facilitate SGBV‐related data collection (Bartels et al., 2019; Mishori et al., 2017). Though they serve as a springboard for studying how ICT can facilitate forensic and testimonial data collection, more impact research is needed to understand how data collection can help lead to better prosecution of perpetrators and ensure the stronger enforcement of laws (which in turn can become deterrents for future SGBV offenses). As such, it is critical to create and analyze valid Theories of Change that link ICT‐based data collection with better enforcement of laws and prosecutions of perpetrators.

Other gaps in the research of ICT for SGBV prevention and/or response interventions in LMIC include, but are not limited to:

- Studies stating a clear theoretical basis and Theory of Change for the intervention, both deemed essential criteria by practitioners and academics for a study of an intervention to be characterized as methodologically rigorous.
- Studies examining how ICT can be used for behavior, attitude and social norm changes.
- Studies examining how access to reliable and comprehensive data (collected and analyzed with ICT modalities) can contribute to SGBV prevention and/or response.
- Studies collecting detailed demographic information that can be used to adapt interventions proven effective in one context to another.
- Studies that explore local contexts and describe local ICT ecosystems in better detail in LMIC.
- Studies that describe and systematically evaluate implementation experiences in LMIC contexts.
- Studies that examine the routine monitoring and evaluating (M&E) of ICT‐based interventions in LMIC, especially in conflict‐related or displacement settings.
- More studies examining how successful evidence‐based eHealth/mHealth interventions connected to global health areas such as HIV and AIDS and reproductive health can be adapted, leveraged, or synthesized to prevent and respond to SGBV.
- Studies examining how ICT interventions can be scaled and sustained, a clear issue that was uncovered in the Landscape Review (Mechael et al., 2021). Without measures to ensure sustainability (e.g., formative appraisals of feasibility and usability), interventions generally are difficult to continue post pilot periods.

### 6.4 Potential biases in the mapping process

As authors of this EGM, we made conscious effort to reduce risk of bias wherever possible. We developed the EGM's protocol, methodology, search strategy, data extraction and analysis under the guidance of a steering committee of diverse SGBV experts and stakeholders with expertise in ICT‐based interventions and LMIC contexts. The steering committee gave continuous feedback regarding the direction of the EGM, including potential areas of bias. Searches and data extraction were undertaken by two independent researchers with discrepancies resolved by discussion to avoid researcher bias. We sought to be comprehensive in the employment of search terms and database selection. We do not claim that this EGM is totally free of limitation or bias, however. While searches were exhaustive, our searches were limited to English, Spanish and Portuguese (our spoken languages). We may have missed evidence published in other languages. While we reviewed all major scientific and academic databases, we may have also missed evidence arquired in smaller, country‐specific, or more niche‐specific databases. Given the relative scarcity of ICT‐based interventions for SGBV in LMIC, we feel confident that the evidence in this EGM is comprehensive. The Landscape Review completed in tandem with this EGM also confirmed the scarcity of evidence for ICT‐based interventions in LMIC outside of the context of peer‐reviewed content.

### 6.5 Limitations of the EGM

The primary limitation to the EGM is the death of methodologically rigorous studies examining the impact of the use of ICT to prevent or respond to SGBV against either women or children in LMIC.

### 6.6 Stakeholder engagement throughout the EGM process

There were no substantive changes in the stakeholder engagement regarding the Steering Committee throughout the EGM process. The authors also conducted an online workshop with 24 SGBV and ICT stakeholders in July 2021 to elicit input into a conceptual framework, and for framing a “Call to Action” for methodologically rigorous research into the use of ICT for prevention of and/or response to SGBV against women and children in LMIC.

## 7 AUTHORS' CONCLUSIONS

### 7.1 Implications for research, practice, and/or policy

This review shows that there is a scarcity of methodologically rigorous studies reporting on the use of ICT in LMIC for the prevention and response to SGBV against women and children. This includes studies ensuring the participation of at least one LMIC‐based researcher as an author.

Our landscape review (separate from this EGM), completed in tandem with this EGM, demonstrated a lgrowing number of ICT‐based programs and interventions for the prevention of, and response to SGBV, in LMIC. However, these have not yet been rigorously evaluated. Future research is needed to better locate, document, and most importantly, evaluate the effectiveness and impact of using ICT for SGBV prevention and response in LMIC contexts.

Most striking is the paucity of studies examining the use of ICT in connection with preventing or responding to SGBV (CSA) against children. In light of the exponential increase in the use of ICT by children and adolescents, even in LMIC, greater attention should be given to examining how ICT can be used during adolescence to address gender norms that lead to SGBV, taking into account the challenges in securing IRB approval for studies in which children are subjects. Ongoing efforts to study and pilot gaming apps and how they can lead to changes in gender norms related to SGBV are promising for preventing future SGBV. (BREAKAWAY Guatemala Pilot Study).^2^ Current efforts to study digital parenting apps can also contribute to developing evidence of using ICT to prevent and respond to CSA (Digital Parenting Studies). Research to study the explicit correlation, if any, of the use of ICT for any of the other evidence‐based strategies identified under the RESPECT and INSPIRE frameworks would also significantly contribute to an evidence base that would inform the use of ICT for SGBV prevention and/or response in LMIC.

While no “clusters” of evidence emerged, several individual studies point to potential emerging areas of future research, including: (1) how ICT can contribute changing gender and social norms related to SGBV and primary prevention; (2) mobile phone applications that promote safety and security; (3) mobile technology for the collection and analysis of survivors' experience with SGBV response services (to inform the design of effective SGBV services); and (4) digital tools that support the collection of forensic evidence for SGBV response and secondary prevention.

The Decker, Wood, Kennedy, et al. (2020) and Decker, Wood, Hameeduddin, et al. (2020) protocol and study around safety decision apps demonstrate how an effective ICT intervention for preventing and of responding to SGBV in an HIC context can be adapted for an LMIC context. Similar efforts to first identity effective interventions in HIC contexts, and then adapt them to LMIC contexts could prove effective in preventing and or responding to SGBV in LMIC.

We also observed a lack of harmonized global impact indicators which further impedes evidence synthesis and overall generalization. Several of the systematic reviews commented on this lack of uniform outcomes and indicators for SGBV and cited this gap as the reason for not completing comprehensive meta‐analysis. Future research is needed to understand how the complexity of SGBV may be uniformly measured across outcomes and indicators, especially within the context of ICT‐based interventions for SGBV response and prevention. Part of this effort includes articulating, understanding, and testing a clear Theory of Change and theoretical basis with each intervention.

In conclusion, ICT‐based interventions contributing to the response and prevention of SGBV in LMIC is an emerging field of study. While there is some very limited evidence showing promise for ICT‐based interventions for SGBV prevention and/or response in LMIC, much more methodologically rigorous research is needed to identify how effective they are, and just what roles they can contribute to preventing and responding to SGBV. To address the gaps in the evidence and build towards a more cohesive evidence base, standard metrics are needed to evaluate the intermediary outcomes aligned to evidence‐based frameworks such as RESPECT and INSPIRE. As more ICT interventions are used for prevention of, and/or response to SGBV, and with the use standard metrics to evaluate their impact, it is anticipated that clusters of evidence will emerge. There are clear examples of interventions that are designed using evidence‐based frameworks that are well‐positioned for evaluation. They are from higher‐income settings that have been rigorously evaluated that are relevant for LMICs, namely safety decision apps and interventions focusing on changing harmful gender‐norms that contribute to SGBV, particularly IPV. Future feasibility and adaptability (from HIC to LMIC) studies will also help researchers and policy‐makers better understand best practice for adapting ICT‐ based interventions for a variety of contexts in LMIC. Such studies will also contribute to the scale‐up and sustainability of interventions, issues that are commonly overlooked.

## CONTRIBUTIONS OF AUTHORS

- Content: William Philbrick, Jacob Milnor, Madhu Deshmukh, Patricia Mechael
- EGM methods: William Philbrick, Jacob Milnor,
- Information retrieval: William Philbrick, Jacob Milnor

## DECLARATIONS OF INTEREST

The project to develop the EGM is funded by a non‐for‐profit organization with no competing interest. The funder is not involved in editorial decisions or decisions related to publishing.

## PLANS FOR UPDATING THE EGM

The project currently has no plans for updating the EGM subsequent to publication.

## DIFFERENCES BETWEEN PROTOCOL AND REVIEW

No substantive changes from the original protocol were made other than amending the Coding Sheet (to include a category for tracking whether any authors were based in LMIC), and a slight change to the title. Further, eligibility for including systematic reviews included a stipulation that at least one of the studies included in a Review was conducted in an LMIC context.

## PUBLISHED NOTES

**Characteristics of studies**

**Characteristics of included studies**

**Table:** 

Ampt et al., 2017
**Notes**
Ampt et al., 2020
**Notes**
Ampt et al., 2020b
**Notes**
Anderson, McClelland, et al., 2019
**Notes**
Anderson, Krause, et al., 2019
**Notes**
Bartels et al., 2019
**Notes**
Brody et al., 2018
**Notes**
Brody et al., 2021
**Notes**
Clark et al., 2020
**Notes**
Clark et al., 2017
**Notes**
Decker, Wood, Kennedy, et al., 2020
**Notes**
Decker, Wood, Hameeduddin, et al., 2020
**Notes**
Eisenhut et al., 2020
**Notes**
Hayes, 2014
**Notes**
Linde et al., 2020
**Notes**
Mishori et al., 2017
**Notes**

**Characteristics of excluded studies**

**Table:** 

Almeida et al., 2018	
**Reason for exclusion**	HIC (Portugal); Conference Paper
Andrade, 2015	
**Reason for exclusion**	HIC
Braithwaite, 2014	
**Reason for exclusion**	HIC
Cardoso and Sorenson, 2017	
**Reason for exclusion**	Good relevant study, but more descriptive. Equated ICT ownership with less likelihood of wife accepting wife beating
Constantino et al., 2015	
**Reason for exclusion**	HIC
Cugelman, 2013	
**Reason for exclusion**	Editorial, not a study
D'Inverno et al., 2016	
**Reason for exclusion**	HIC context
Danis et al., 2018	
**Reason for exclusion**	HIC
Divakar et al., 2019	
**Reason for exclusion**	All HIC studies
Edwards‐Gaura et al., 2014	
**Reason for exclusion**	HIC
El Morr & Layal, 2019	
**Reason for exclusion**	All 25 studies from high income countries
El Morr, 2020	
**Reason for exclusion**	All High Income County
Elia, 2017	
**Reason for exclusion**	Not a study per se; not directly linked to SGBV
Emezue, 2020	
**Reason for exclusion**	More of a discussion/landscapring paper. Not a study, although it cites studies
Estrela et al., 2020	
**Reason for exclusion**	“Technology” does not refer to use of ICT
Ford‐Gilboe, 2017	
**Reason for exclusion**	HIC
Garnweidner‐Holme et al., 2020	
**Reason for exclusion**	HIC
Georgia Salivar et al., 2020	
**Reason for exclusion**	HIC
Gilbert et al., 2016	
**Reason for exclusion**	HIC
Glass, 2017	
**Reason for exclusion**	HIC context
Glass, 2021	
**Reason for exclusion**	HIC context
Grace‐Farfaglia, 2019	
**Reason for exclusion**	HIC, Scoping review
**Hagarty 2019**	
**Reason for exclusion**	HIC
Hawkins et al., 2009	
**Reason for exclusion**	HIC content
Hilton & Ham, 2014	
**Reason for exclusion**	HIC; not an impact study
Jeyarman, 2020	
**Reason for exclusion**	HIC context
Jiménez‐Rodríguez et al., 2020	
**Reason for exclusion**	HIC
Jozkowski & Ekbia, 2015	
**Reason for exclusion**	HIC
Karakurt et al., 2017	
**Reason for exclusion**	HIC
Karystianis et al., 2019	
**Reason for exclusion**	HIC
Konijnendijk et al., 2019	
**Reason for exclusion**	HIC
Koziol‐McLain et al., 2018	
**Reason for exclusion**	HIC
Newins & White, 2021	
**Reason for exclusion**	HIC
Rempel 2019	
**Reason for exclusion**	Scoping/landscape review, not evidence; only online interventions, no apps
Salazar et al., 2014	
**Reason for exclusion**	HIC
Salazar et al., 2017	
**Reason for exclusion**	HIC
Salazar et al., 2019	
**Reason for exclusion**	HIC
Sorbring et al., 2015	
**Reason for exclusion**	HIC
Tait et al., 2019	
**Reason for exclusion**	HIC, alcohol use not connected to SGBV
van Gelder et al., 2020	
**Reason for exclusion**	HIC; still under development; will be trial

**Characteristics of studies awaiting classification**

**Characteristics of ongoing studies**

***BREAKAWAY*** **Guatemala Pilot Study**

**Table:** 

**Study name**	*Using* BREAKAWAY *Mobile Game at Youth Camps to Address Violence Against Women and Girls, a Pilot Project in Guatemala*
**Starting date**	2021
**Contact information**	Wendi Stein: wstein@populationmedia.org
**Notes**	Pilot Study for *BREAKAWAY* gaming app and program with university students and adolescents in Guatemala.

***Digital Parenting Studies***

**Table:** 

**Study name: Parenting for Lifelong Health Digital (PLH Digital)**
**Starting date: 2019**
**Contact information: Lucie Cluver**, lucie.cluver@spi.ox.ac.uk
Notes: The Parenting for Lifelong Health Digital (PLH Digital) project aims to convert the evidence‐based, in‐person version of Parenting for Lifelong Health for Teens (PLH for Teens) into a digital format. The PLH for Teens programme aims to foster better relationships between parents and their children by improving positive parenting skills, improving child behaviour, building social support for parents, strengthening financial coping and reducing parenting stress. After showing positive results in a cluster randomised trial conducted in South Africa with UNICEF and WHO from 2015‐18, the programme has been translated into 16 languages and delivered by NGOs and governments to 180,000 families across 18 low‐resource countries. Despite this rapid scale‐up, the programme only reaches a fraction of those in need as they are currently delivered through in‐person training. PLH Digital therefore aims to scale up the reach and impact of PLH for Teens by developing an app‐based version of the programme. The iterative development of a basic version of the app will be carried out with families in Nigeria, South Africa and Kenya, and with parenting trainers at the NGO, Clowns Without Borders South Africa. The app will be tested in a pan‐African randomised controlled trial to determine its efficacy. https://www.spi.ox.ac.uk/parenting-for-lifelong-health-digital-plh-digital#collapse3697276

## SUMMARY OF FINDINGS TABLES

**Table 2:** Summary of findings: Primary studies

Study	Type of intervention/how ICT used	Outcome measurements	Type of study	Target group	Location/context	Outcome results
Ampt et al. (2020)	Mobile phone SMS messaging to SRH change behavior (“WHISPER”) and nutrition (“SHOUT” ‐ original protocol)	Primary: pregnancy incidence and SRH related outcomes “GBV, stigma and rights” was one of the intervention's “domains” in original protocol. Not mentioned in final study results	2‐arm cluster‐randomized control trial	Women: self‐ reported female sex workers aged 16–34	Mombasa Kenya/self‐reported female sex workers	The intervention had no measurable effect on unintended pregnancy incidence. Mobile health interventions, even when acceptable and rigorously designed, are unlikely to have a sufficient effect on behavior among female sex workers to change pregnancy incidence when used in isolation. No mention of GBV or other originally stated intermediate outcomes including relationship control, health service seeking and utilization in final published study results (No mention in study results connected with nutrition component‐“SHOUT”)
Bartels et al. (2019)	“SenseMaker” survey methodology tool for M&E of GBV programs, generating mixed methods data, delivered through app on a tablet or on a computer/browser	Value of using SenseMaker tool to generate timely mixed methods data on GBV programs and services, to gauge experiences, motivations, perceptions of benefits, and outcomes of accessing such GBV services. (evaluation or more the survey tool rather than the role of ICT)	Mixed‐methods, cross‐sectional M&E study	Women and girls age 11 and over who had accessed GBV‐related program or service	Lebanon (5 locations)/humanitarian	Demonstrated that the single ICT‐based tool, based on location, can be effective in collecting a holistic nuanced insights could lead to difference perceived benefits by type of GBV program, how motivations for accessing programs differed by location, and how feelings while accessing programs differed by nationality. Motivational outcomes for accessing the service (differing in each location) included: protection/security, access to group activities, and financial assistance.
Brody et al. (2021)	“Mobile Link” for improving female entertainment workers' health by connecting them to existing HIV, sexual and reproductive health, and GBV services using short message services (SMS) and voice messages (VM)	Primary: self‐reported HIV and STI testing, condom use, and contraceptive use. Secondary: contact with outreach workers, escorted referral services use, forced drinking, and GBV experience	Randomized controlled trial	Female entertainment/sex workers (ages 18–30)	Cambodia (2 locations)	For reporting of GBV experiences, quantitative findings were not significant; did see an increase in request for GBV services and believing there was something one could do in response to violence. The Mobile Link intervention effectively connected FEWs with outreach workers and escorted referrals. Reductions in forced drinking at work were also significantly more extensive in the intervention group than the control group.
Clark et al. (2020)	Multi‐media social and behavior change strategy involving radio drama, community mobilization with listener engagement through SMS, and Interactive Voice Response (IVR) (radio and phone).	Primary: Physical and/or IPV in prior 12 months Secondary: Psychological partner abuse in prior 12 months Economic partner abuse in prior 12 months Conflict and conflict resolution techniques in prior 12 months Couple communications in prior 12 months Attitudes toward the acceptability of IPV Perceptions of community‐norms on the acceptability of partner violence	Randomized controlled trial and concurrent mixed method design	Random sampling of women, reproductive age (18–49 years old), with husband who was at lease 18 years old, living with their husband most of the year, and husband had no plan to migrate in the coming 2 years.	Nepal	Note that the ICT components were not isolated for evaluation. Both control and intervention groups exposed to radio, SMS and IVR. Accordingly, there are no study conclusions regarding the role or impact that ICT had. The trend in IPV for both groups was nonlinear, largely declining at midline (control condition) and rising again at endline (control and intervention conditions), possibly reflecting greater reporting due to awareness‐raising activities. Significant differences between the two groups were largely absent at endline. Higher listening and discussion attendance was associated with decreases in several forms of IPV, some of which persisted to endline. These findings suggest that intensive community engagement over longer time spans or social network measurement may be necessary to detect significant changes at the community level. While there was little overall mean change from baseline to 28 months after baseline, there was considerable diversity in baseline norms levels. Better targeting the intervention to those areas with especially gender inequitable norms may enhance norms intervention effectiveness. It is clear from this analysis that norms change is possible and most likely to occur in communities where the need is greatest.
Decker, Wood, Hameeduddin, et al. (2020)	Adapted (from HIC for Kenya) safety planning decision algorithm (myPlan). Provides support for defining healthy relationships with descriptions of relationships which are not healthy; safety strategies by helping women identify severity of the violence and potential danger to self and family; decision‐making; and healing via validating messages	Primary: safety preparedness, decisional conflict, use, and helpfulness of strategies, IPV Secondary: resilience, relationship quality, depression, consideration and seeking IPV support services, self‐blame, recognition of abuse, self‐efficacy, and danger score	Randomized controlled trial	Women, age 18+	Kenya (urban)	At 3 months, intervention participants reported increased helpfulness of safety strategies used relative to control participants; IPV reduced in both groups. Among women reporting the highest level of IPV severity, intervention participants had significant increase in resilience compared with controls, and significantly decreased risk for lethal violence. Facilitated delivery of a technology‐based safety intervention appropriately adapted to the context demonstrates promise in improving women's IPV‐related health and safety in a low‐resource, urban setting.
Mishori et al. (2017)	Application (MediCapt)that uses ICT (1) to enable health care providers to gather and compile medical evidence related to sexual violence in a standardized manner, and (2) to securely transmit the evidence to authorities engaged in prosecuting and seeking accountability for such crimes (such as investigating officers, gender desk officers in the police force)	Usability, Acceptability; feasibility and sustainability Not an impact or outcome study; but rather a description of a participatory development and user design process with Congolese end‐users of a novel human rights app for clinicians intended to standardize the documentation of sexual violence evidence for forensic and legal purposes.	Survey questions, key informant interviews, and focus group discussions		DRC (post‐conflict)/humanitarian	Describes the preliminary user feedback on a mobile app in development, MediCapt, to document sexual violence evidence for forensic and legal purposes. To assess the efficacy and effectiveness of the app, a robust monitoring and evaluation process has been developed and will be carried out longitudinally. Determining the true impact of MediCapt on medical, legal, and human rights outcomes will require years of study and certain methodological revisions

**Table 3:** Summary of findings: “Systematic” reviews (e.g., multiple study reviews)

Title	Outcome	# of included studies	# of studies from LMIC	AMSTAR risk of bias	Notes
Anderson et al. (2019)	IPV prevention	31	1	Low	Included the Brody et al. (2018) (protocol) for Brody et al. (2021)
Eisenhut et al. (2020)	Mobile applications addressing violence against women	171	95	Risk of bias present	This review did not look at peer‐reviewed studies but rather a landscape of mobile applications for VAW. The review did not include an actual list of those included individual applications.
Hayes (2014)	Mobile and internet technologies for gender‐based violence	7	7	Not systematic	This review is a landscape review of several ICT‐based projects in the LMIC. It did not include any peer‐reviewed studies but reviewed existing programs for SGBV with a tech‐facilitated intervention.
Linde et al. (2020)	IPV	14	2	Low	Included Clark et al. (2020) and a trial registration from Kenya

## SOURCES OF SUPPORT

**Internal sources**

- N/A

**External sources**

- The project to develop the EGM is funded by a non‐for‐profit organisation with no competing interest. The funder is not involved in editorial decisions or decisions related to publishing.

## Supporting information

Supporting information.

## Footnotes

^1^
SGBV, sometimes referred to as gender‐based violence (GBV) is “any harmful act of sexual, physical, psychological, mental, and emotional abuse that is perpetrated against a person's will and that is based on socially ascribed (i.e., gender) differences between males and females” United Nations Office for the Coordination of Humanitarian Affairs (UNOCHA, 2019).

^2^
We identified an on‐going study in Guatemala concerning a gaming app, *BREAKAWAY*. While this study is only at initiation as of writing our review, it explores harmful gender norms through the use of a phone and tablet‐based game, using a football‐related narrative. It is the only example of primary prevention that we identified in LMIC.
