# Supplementary material for: Information and communications technology use to prevent and respond to sexual and gender‐based violence in low‐ and middle‐income countries: An evidence and gap map
Source: Campbell Syst Rev. 2022 Oct 25;18(4):e1277. doi: 10.1002/cl2.1277 (PMC9595343; doi:10.1002/cl2.1277)
Supplement: Supplementary file 1 — Supporting information. [file CL2-18-e1277-s001.docx]

# Appendices

## 1 Link to online interactive EGM

Add link at the full report stage

## 2 Table of Illustrative Interventions

Table of Illustrative Interventions

| **Intervention Objective (Intermediate Result or Primary Outcome):** | **ICT Intervention** | **Targeted Group(s) (Primary or Intermediate)** | **Outcome/Impact** |
| --- | --- | --- | --- |
| Increase in knowledge of physical locations of high SGBV incidents  to avoid (Primary). | Mobile SMS  and web-based mapping application alerting of high SGBV risk locations | Women (primary) | Secondary  Prevention: reduction in incidence of SGBV in certain locations |
| Mobile phone or web-based referrals to SGBV service providers (Intermediate) | Mobile phone referrals applications | Service providers (intermediate) | Improve access to services; including prevention services: increase in number or proportion of SGBV survivors who access SGBV service providers |
| Provide children access to SGBV counselling services (Primary)  Prevent further violence to children (Intermediate) | Childline/Child Helpline mobile phone counselling | Children (primary) | Improve access to services & Tertiary prevention: increase in number of children who access services; decrease in number/proportion of SGBV child survivors who experience a re-occurrence of SGBV |
| Change social norms around violence between partners (intermediate) | Mobile phone messaging around masculinity and violence | Intimate Partners (intermediate) | Primary prevention & Secondary prevention: Reduction of number or rate of SGBV incidents |
| Changing social norms around SBGV against children (prevention)  Educating about how to best respond to SBGV (response) | Gaming applications on mobile phones and tablets | Children and adolescents, women (primary and intermediate) | Primary, Secondary and Tertiary prevention; improve access to services: reduction of number or rate of SGBV incidents and/or reoccurrence |

## 3 Coding Sheet Examples

**Coding Sheet Examples**

| **1: Study Characteristics** | **Definition** | **Example** |
| --- | --- | --- |
| 1.1: Organization | 1=University, 2=Government Entity, 3=Contract Research Firm, 4=NGO, 5=IGO, 6=Civil Society, 7=Mixed, 8=Other (specify) | (4) CARE |
| 1.2: Publication Language | 1=English, 2=Portuguese, 3=Spanish |  |
| 1.3: Methods Used | 1=Quantitative, 2=Qualitative, 3=Mixed-methods, 4=other (specify) |  |
| 1.4: Participatory Design | 1=Yes, 2= No |  |
| 1.5: Theoretical Framework Used | 1=Yes (specify), 2=No | (1) RESPECT Framework |
| 1.6: Study Duration | 1=One Day or Less, 2=One Week or Less, 3=One Month or Less, 4=One Marking Period or Less, 5=One Semester or Less, 6=One Year or Less, 7= More Than One Year |  |
| 1.7: Control Group | 1=Yes, 2=No |  |

| **2: Geographics** | **Definition** | **Example** |
| --- | --- | --- |
| 2.1: Region | 1=Africa, 2=Asia, 3=Central America, 4=Europe, 5=North America, 6=South America, 7=Southeast Asia |  |
| 2.2: Country Income | 1=Lower Income Country, 2=Middle Income Country, 3=High Income Country |  |
| 2.3: Country (Name) | (specify) | South Africa |

| **3: Population Characteristics** | **Definition** | **Example** |
| --- | --- | --- |
| 3. 1: Target Population | 1=Women Only (18+), 2=Girls Only (<18), 3= Women and Girls, 4=Men (18+), 5=Boys Only (<18), 6=Boys and Men, 7=Children (Boys and Girls <18), 8=LGBTQ, 9=First Responders, 10=Other (specify) | (2) Girls Age 12-17 |
| 3.2: Key populations | 1=Yes (specify) 2=No. | (1) HIV positive populations |
| 3.3: Environment | 1=Urban, 2=Rural, 3=Peri-urban, 4=Mixed, 5=Not Specified |  |
| 3.4: Setting/Context | 1= Extreme Poverty, 2=Natural Emergency/Disaster, 3=Armed forces, 4=Conflict/post conflict, 5= trafficking, 6= Migration, 7=IDP/Refugee, 8=School, 9=Sex work, 10= Medical, 11= Social Services, 12= Other (specify) | (8) Children in Secondary Schools in Sao Paulo, Brazil. |
| 3.5: Sectors Implicated | 1=Health, 2= Justice/legal, 3=social welfare, 4= Education, 5=Other, 6= Coordination across sectors | (1) Frontline community healthcare workers in Sub-Saharan Africa |

| **4: ICT/Intervention Design** | **Definition** | **Example** |
| --- | --- | --- |
| 4.1: SGBV Prevention or Response | 1=Prevention and/or 2= Response | (2) Linkage to first response services |
| 4.2: ICT Medium | 1= Mobile Phone, 2=Tablet, 3 = Web-Based Laptop or PC, 4=Podcast, 5=Other (specify) | (1) Mobile Phone application |
| 4.3: Intervention Type | 1=Games/Gaming, 2=Education/Awareness , 3=Referrals, 4=Reporting,  5= Case Management, 6=Training, 9=Access to Services, 10=Community Mobilization, 11=Data Collection and Management,  12= Counseling,13= Diagnostics; 14=Messaging; 15=Decision Making Protocols; 16= Mapping; 17=Entertainment | (5) Mobile Phone App to Facilitate Case Management for IPV Survivors |
| 4.5: Intervention Sustainability Addressed | 1=Yes, 2=No | (2) ICT Implementation Plan Does Not Include Provision for future financing |

| **5: Outcomes** | **Definition** | **Example** |
| --- | --- | --- |
| 5.1: Outcome Target | 1=Individual 2= Interpersonal, 3= Community, 4= Societal | (4) Increased IPV Reporting |
| 5.2: Outcome Scope | 1= Relationship Skills Strengthened, 2= Empowerment of Women (or target group); 3=Services Access Ensured; 4=Poverty Reduction; 5= Safe Environments; 6= Prevention of Child and Adolescent Abuse; 7= Improved SGBV Attitudes, Beliefs, Norms; 8= Parent or Caregiver Support; 9= Implementation and Enforcement of Laws, 10= Diagnostics, 11= Response Services Strengthened | (5) School Teachers Trained on Recognizing SGBV Risk in Adolescent Girls at Secondary Schools, and taking appropriate actions |
| 5.2: Impact Measured | Y=Yes, N=No  1= Level of SGBV; 2= Referrals; 3= Access to Services | Y(1) National SGBV Incidence |
| 5.3: Includes Prevention of SGBV Recurrence | 1=Yes, 2=No, 3=N/A | (1) First Response with Linkage to Follow Up Social Services and judicial protection |
